# Synthesis of cross-conjugated trienes by rhodium-catalyzed dimerization of monosubstituted allenes

**DOI:** 10.3762/bjoc.7.67

**Published:** 2011-05-09

**Authors:** Tomoya Miura, Tsuneaki Biyajima, Takeharu Toyoshima, Masahiro Murakami

**Affiliations:** 1 Department of Synthetic Chemistry and Biological Chemistry, Kyoto University, Katsura, Kyoto 615-8510, Japan

**Keywords:** allene, cross-conjugated triene, dimerization, rhodium, stereoselective

## Abstract

A rhodium(I)/dppe catalyst promoted dimerization of monosubstituted allenes in a stereoselective manner to give cross-conjugated trienes, which are different from those obtained by a palladium catalyst.

## Introduction

**Scheme 1:**
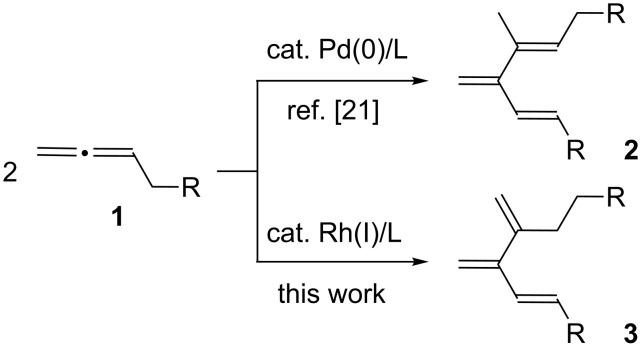
A new entry to substituted cross-conjugated trienes.

Cross-conjugated trienes, known as [3]dendralenes [1], are attractive synthetic precursors used for consecutive double [4 + 2] cycloaddition reactions [2–4] to provide a rapid access to polycyclic carbon frameworks. Thus, a number of methods for the preparation of the parent 3-methylenepenta-1,4-diene [5] and its substituted derivatives [6–17] has been developed. Among these, transition-metal-catalyzed dimerization of allenes presents a unique entry to substituted cross-conjugated trienes. For example, a nickel(0)/triphenylphosphine complex catalyzes a dimerization reaction of 3-methylbuta-1,2-diene to afford 2,5-dimethyl-3,4-bismethylenehex-1-ene [18–19]. The nickel-catalyzed reaction, however, leads to a complex mixture of products when monosubstituted allenes such as penta-1,2-diene and 1-phenylpropa-1,2-diene are employed [20]. On the other hand, a palladium-catalyzed dimerization reaction of monosubstituted allenes produces substituted cross-conjugated trienes **2** in high yield (Scheme 1) [21]. We report here that dimerization of monosubstituted allenes is also catalyzed by a rhodium(I)/dppe complex to form cross-conjugated trienes **3**, which are different from those obtained with the palladium catalyst.

## Results and Discussion

We initiated our study using undeca-1,2-diene (**1a**) as the model substrate and a rhodium(I) complex as the catalyst (Table 1). When **1a** was treated with a catalytic amount of [RhCl(cod)]_2_ (2.5 mol %, cod = cycloocta-1,5-diene) in toluene at 130 °C for 12 h, **2a** was formed in 40% NMR yield along with another minor dimerized product (13% NMR yield) and unidentified compounds (Table 1, entry 1). The structure of the minor dimerized product was determined to be (*E*)-10,11-dimethyleneicos-8-ene (**3a**) by ^1^H and ^13^C NMR spectroscopy. Thus, the two isomeric dimers, one identical to the isomer obtained by the palladium-catalyzed reaction and the other a different isomer, were produced by the rhodium-catalyzed reaction. Next, several phosphine ligands were examined (Table 1, entries 2–5). To our delight, the use of the dppe ligand suppressed the formation of **2a** and the unidentified compounds, and increased the NMR yield of **3a** to 96% (86% isolated yield, Table 1, entry 4). A complex mixture of products was obtained when the reaction temperature was lowered from 130 °C to 90 °C (Table 1, entry 6). Moreover, the use of [Rh(OH)(cod)]_2_ and Rh(acac)(cod) as the precatalyst resulted in a decrease of the reaction rate (Table 1, entries 7 and 8).

**Table 1:** Optimization of reaction conditions^a^.

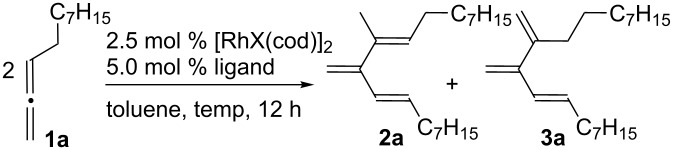					

Entry	X	Ligand^b^	*T* (°C)	Yield of **2a** (%)^c^	Yield of **3a** (%)^c^

1	Cl	none	130	40	13
2	Cl	PPh_3_^d^	130	24	18
3	Cl	dppm	130	24	37
4	Cl	dppe	130	<5	96 (86)
5	Cl	dppp	130	17	50
6	Cl	dppe	90	38	24
7	OH	dppe	130	40	10
8	acac^e^	dppe	130	44	<5

^a^Reactions conducted on a 0.4 mmol scale. ^b^dppm = 1,1-bis(diphenylphosphino)methane, dppe = 1,2-bis(diphenylphosphino)ethane, dppp = 1,3-bis(diphenylphosphino)propane. ^c^NMR yield using mesitylene as an internal standard. Isolated yield given in parenthesis. ^d^Using 10 mol % of PPh_3_. ^e^Using 5.0 mol % of Rh(acac)(cod).

**Scheme 2:**
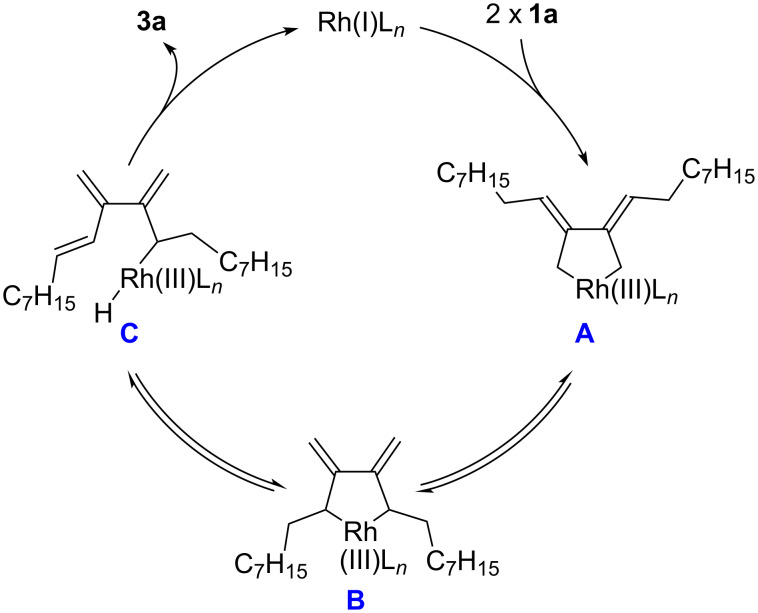
A proposed reaction pathway.

We propose that the dimerization reaction proceeds through the pathway outlined in Scheme 2. Initially, two molecules of **1a** coordinate to a rhodium(I) center at the terminal carbon–carbon double bonds from their sterically less-hindered sides. Oxidative cyclization occurs in a head-to-head manner to form the five-membered rhodacyclic intermediate **A** [22–25], which is in equilibrium with another rhodacyclic intermediate **B** via σ–π–σ isomerization. Then, β-hydride elimination takes place with **B** to form rhodium hydride **C** stereoselectively. Finally, reductive elimination from **C** yields **3a** together with the catalytically active rhodium(I) complex. It is also conceivable, however, that oxidative cyclization of two molecules of **1a** occurs in a tail-to-tail manner to directly furnish **B**. The other isomer **2a** could be formed through allylic 1,3-migration of rhodium from **C** and subsequent reductive elimination.

Under the optimized reaction conditions using dppe as the ligand, various monosubstituted allenes **1b**–**j** were subjected to the catalytic dimerization reaction (Table 2). In most cases, essentially one isomer **3** was formed, and the other isomer **2** was barely detectable in the ^1^H NMR spectrum of the crude reaction mixture (<5%). Allenes **1b**–**i** possessing a primary alkyl group reacted well to afford the corresponding products **3b**–**i** in yields ranging from 63% to 90% (Table 2, entries 1–8). Functional groups such as benzyloxy, siloxy, hydroxy and cyano groups were tolerated in the alkyl chain under the reaction conditions. Cyclohexylpropa-1,2-diene (**1j**) possessing a secondary alkyl group also participated in the dimerization reaction (Table 2, entry 9). On the other hand, 1,1-disubstituted allenes such as 3-methylbuta-1,2-diene and 3-pentylocta-1,2-diene failed to undergo the dimerization reaction, in contrast to the nickel-catalyzed reaction [18–19].

**Table 2:** Synthesis of cross-conjugated trienes by the allene dimerization reaction^a^.

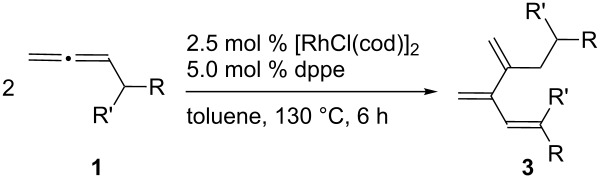					

Entry	**1**	R	R'	**3**	Yield (%)^b^

1	**1b**	C_5_H_11_	H	**3b**	85
2	**1c**	CH_2_Ph	H	**3c**	78^c^
3	**1d**	Cy	H	**3d**	83^d^
4	**1e**	CH_2_OBn	H	**3e**	70
5	**1f**	(CH_2_)_3_OBn	H	**3f**	78
6	**1g**	(CH_2_)_3_OSiMe_2_*t*-Bu	H	**3g**	90
7	**1h**	(CH_2_)_3_OH	H	**3h**	63
8	**1i**	(CH_2_)_3_CN	H	**3i**	75
9	**1j**	–(CH_2_)_5_–		**3j**	60^d^

^a^Reactions conducted on a 0.4 mmol scale. ^b^Isolated yield unless otherwise noted. ^c^The product was accompanied by a small amount of an unidentified impurity. ^d^NMR yield using mesitylene as an internal standard.

**Scheme 3:**
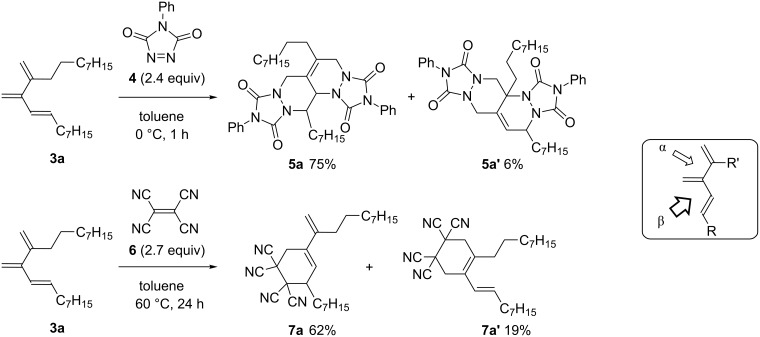
[4 + 2] cycloaddition reaction of **3a** with PTAD and TCNE.

Next, we examined the consecutive double [4 + 2] cycloaddition reaction of the cross-conjugated trienes obtained in the present study. Triene **3a** was treated with 4-phenyl-1,2,4-triazoline-3,5-dione (**4**, PTAD), a highly reactive dienophile, in toluene at 0 °C (Scheme 3). The conversion of **3a** was complete within 1 h, and after chromatographic isolation, bisadducts **5a** and **5a’** were obtained in 75% and 6% yields, respectively. The major bisadduct **5a** resulted from initial addition to the more congested diene moiety of **3a** (site β). When tetracyanoethylene (**6**, TCNE), which was a less reactive dienophile than **4**, was used, [4 + 2] cycloaddition also occurred preferentially at site β, but only once on heating at 60 °C for 24 h.

## Conclusion

In summary, we have developed a new dimerization reaction of monosubstituted allenes catalyzed by a rhodium(I)/dppe complex, allowing the stereoselective formation of substituted cross-conjugated trienes. It is interesting that the rhodium catalyst and the palladium catalyst gave different types of cross-conjugated trienes.

## Experimental

### General procedure for rhodium-catalyzed dimerization of monosubstituted allenes

To a side-arm tube equipped with a stirrer bar, was added [RhCl(cod)]_2_ (4.9 mg, 2.5 mol %) and dppe (7.7 mg, 5 mol %). The tube was evacuated and refilled with argon three times. Then, toluene (4 mL) and substrate **1** (0.4 mmol) were added via syringe and the tube was closed. After heating at 130 °C for 6 h, the reaction mixture was cooled to room temperature, passed through a pad of Florisil^®^ and eluted with ethyl acetate (≈ 90–100 mL). The filtrate was concentrated under reduced pressure and the residue purified by preparative thin-layer chromatography to give product **3**. Although the isolated **3** was relatively labile, it could be kept at −30 °C for days without any detectable decomposition or polymerization.

## Supporting Information

File 1 Experimental details and spectroscopic data for new compounds.
