# Supplementary material for: Synthesis of cross-conjugated trienes by rhodium-catalyzed dimerization of monosubstituted allenes
Source: Beilstein J Org Chem. 2011 May 9;7:578–81. doi: 10.3762/bjoc.7.67 (PMC3107559; doi:10.3762/bjoc.7.67)
Supplement: File 1 — Experimental details and spectroscopic data for new compounds. [file Beilstein_J_Org_Chem-07-578-s001.pdf]

Supporting Information
for
Synthesis of cross-conjugated trienes by rhodium-catalyzed dimerization of monosubstituted allenes

Tomoya Miura, Tsuneaki Biyajima, Takeharu Toyoshima and Masahiro Murakami*

Address: Department of Synthetic Chemistry and Biological Chemistry,

Kyoto University, Katsura, Kyoto 615-8510, Japan

Email: Tomoya Miura - tmiura@sbchem.kyoto-u.ac.jp; Masahiro Murakami* - murakami@sbchem.kyoto-u.ac.jp

* Corresponding author

Experimental details and spectroscopic data for new compounds.

Table of Contents:

S2	General methods and materials
	Spectroscopic data (**1h**, **1i**)
	General procedures for rhodium-catalyzed dimerization reaction of monosubstituted allenes **1**
	Spectroscopic data (**3a**, **3b**)
S3	Spectroscopic data (**3c**, **3d**, **3e**, **3f**, **3g**, **3h**, **3i**, **3j**)
S4	Procedure for Diels–Alder reaction of *N*-phenyl-1,2,4-triazoline-3,5-dione (**4**) with **3a**
	Spectroscopic data (**5a**, **5a'**)
	Procedure for Diels–Alder reaction of tetracyanoethylene (**6**) with **3a**
	Spectroscopic data (**7a**, **7a'**)
	References
S5–S6	^{1}H and ^{13}C NMR spectra of **1h**
S7–S8	^{1}H and ^{13}C NMR spectra of **1i**
S9–S10	^{1}H and ^{13}C NMR spectra of **3a**
S11–S12	^{1}H and ^{13}C NMR spectra of **3b**
S13–S14	^{1}H and ^{13}C NMR spectra of **3c**
S15–S16	^{1}H and ^{13}C NMR spectra of **3d**
S17–S18	^{1}H and ^{13}C NMR spectra of **3e**
S19–S20	^{1}H and ^{13}C NMR spectra of **3f**
S21–S22	^{1}H and ^{13}C NMR spectra of **3g**
S23–S24	^{1}H and ^{13}C NMR spectra of **3h**
S25–S26	^{1}H and ^{13}C NMR spectra of **3i**
S27–S28	^{1}H and ^{13}C NMR spectra of **3j**
S29–S30	^{1}H and ^{13}C NMR spectra of **5a**
S31–S32	^{1}H and ^{13}C NMR spectra of **5a'**
S33–S34	^{1}H and ^{13}C NMR spectra of **7a**

General Methods: All reactions were carried out under an argon atmosphere unless otherwise noted. Infrared spectra were recorded on a Shimadzu FTIR DR-8000 spectrometer. ^{1}H and ^{13}C NMR spectra were recorded on a Varian Mercury vx400 (^{1}H at 400 MHz and ^{13}C at 100 MHz) spectrometer using $CHCl_3$ (^{1}H, δ = 7.26) and $CDCl_3$ (^{13}C, δ = 77.0) as an internal standard unless otherwise noted. High-resolution mass spectra were recorded on a JEOL JMS-SX102A (EI), a Thermofisher OrbitrapXL (ESI, APCI corona), or JMS-HX110A (FAB) spectrometer. Flash column chromatography was performed with silica gel 60 N (Kanto). Preparative thin-layer chromatography was performed on silica gel plates with PF254 indicator (Merck).

Materials: Toluene was distilled from sodium/benzophenone ketyl. $[RhCl(cod)]_2$ was prepared according to the literature procedure [1]. 1,2-Bis(diphenylphosphino)ethane (TCI) was used as received from commercial sources. Undeca-1,2-diene (**1a**) [2], nona-1,2-diene (**1b**) [2], penta-3,4-dien-1-ylbenzene (**1c**) [2], buta-2,3-dien-1-ylcyclohexane (**1d**) [2], 5-bezyloxy-penta-1,2-diene (**1e**) [3], 7-benzyloxy-hepta-1,2-diene (**1f**) [3], 7-(*tert*-butyldimetylsiloxy)-hepta-1,2-diene (**1g**) [3], hepta-5,6-dien-1-ol (**1h**) [3], octa-6,7-dienenitrile (**1i**) [3], propa-1,2-dien-1-ylcyclohexane (**1j**) [2] were prepared according to the literature procedures.

1h [4]: IR (neat): 3314, 2934, 2860, 1956, 1435 cm^{-1}; ^{1}H NMR: 1.36 (br s, 1H), 1.43–1.54 (m, 2H), 1.56–1.68 (m, 2H), 1.98–2.08 (m, 2H), 3.60–3.70 (m, 1H), 4.66 (dt, *J* = 6.8, 3.2 Hz, 2H), 5.10 (quint, *J* = 6.8 Hz); ^{13}C NMR: 25.1, 27.9, 32.1, 62.8, 74.8, 89.7, 208.5; HRMS (APCI corona): Calcd for $C_7H_{13}O$, M+H$^+$ 113.0966. Found *m/z* 113.0964.

1i: IR (neat): 2937, 2860, 2245, 1956, 1425 cm^{-1}; ^{1}H NMR: δ = 1.52–1.64 (m, 2H), 1.65–1.76 (m, 2H), 2.00–2.08 (m, 2H), 2.35 (t, *J* = 7.2 Hz, 2H), 4.68 (dt, *J* = 6.8, 3.2 Hz, 2H), 5.08 (quint, *J* = 6.8 Hz, 1H); ^{13}C NMR: δ = 16.9, 24.6, 27.2, 27.7, 75.3, 88.9, 119.7, 208.5; HRMS (ESI$^+$): Calcd for $C_8H_{11}NNa$, M+Na$^+$ 144.0789. Found *m/z* 144.0784.

General procedures for the rhodium-catalyzed dimerization of monosubstituted allenes 1.
To a side-arm tube equipped with a stirrer bar, was added $[RhCl(cod)]_2$ (4.9 mg, 2.5 mol %) and dppe (7.7 mg, 5 mol %). The tube was evacuated and refilled with argon three times. Then, toluene (4mL) and substrate **1** (0.4 mmol) were added via syringe and the tube was closed. After heating at 130 °C for 6 h, the reaction mixture was cooled to room temperature, passed through a pad of Florisil(R) and eluted with ethyl acetate (ca 90–100mL). The filtrate was concentrated under reduced pressure and the residue purified by preparative thin-layer chromatography to give product **3**. Although the isolated **3** was relatively labile, it could be kept a −30 °C for days without any detectable decomposition or polymerization.

3a: IR (neat): 2924, 2853, 1463 cm^{-1}; ^{1}H NMR: δ = 0.88 (t, *J* = 7.2 Hz, 6H), 1.18–1.46 (m, 24H), 2.09 (q, *J* = 7.2 Hz, 2H), 2.20 (t, *J* = 7.2 Hz, 2H), 4.89 (d, *J* = 1.6 Hz, 1H), 4.95 (s, 2H), 4.98 (s, 1H), 5.74 (dt, *J* = 15.6, 6.8 Hz, 1H), 6.06 (d, *J* = 15.2 Hz, 1H); ^{13}C NMR: δ = 14.09, 14.11, 22.67, 22.69, 28.2, 29.2, 29.3, 29.35, 29.38, 29.5, 29.6, 29.7, 31.8, 31.9, 32.8, 35.4, 112.1, 112.8, 130.3, 133.3, 148.5, 148.9; HRMS (EI$^+$): Calcd for $C_{22}H_{40}$, M$^+$ 304.3130. Found *m/z* 304.3138.

3b: IR (neat): 2925, 2855, 1467 cm^{-1}; ^{1}H NMR: δ = 0.88 (t, *J* = 6.8 Hz, 3H), 0.89 (t, *J* = 6.4 Hz, 3H), 1.20–1.46 (m, 16H), 2.09 (q, *J* = 7.2 Hz, 2H), 2.20 (t, *J* = 7.2 Hz, 2H), 4.90 (s, 1H), 4.95 (s, 2H), 4.98 (s, 1H), 5.74 (dt, *J* = 15.6, 6.8 Hz, 1H), 6.06 (d, *J* = 15.6 Hz, 1H); ^{13}C NMR: δ = 14.06, 14.11, 22.5, 22.7, 28.2, 28.9, 29.2, 29.3, 31.4, 31.9, 32.8, 35.4, 112.1, 112.8, 130.3, 133.4, 148.4, 148.9; HRMS (EI$^+$): Calcd for $C_{18}H_{32}$, M$^+$ 248.2504. Found *m/z* 248.2494.

3c: IR (neat): 3025, 2936, 1495, 1452 cm^{-1}; ^{1}H NMR: δ = 1.74 (quint, *J* = 7.6 Hz, 2H), 2.26 (t, *J* = 7.6 Hz, 2H), 2.60 (t, *J* = 7.6 Hz, 2H), 3.44 (d, *J* = 6.8 Hz, 2H), 4.95 (d, *J* = 2.0 Hz, 1H), 4.97–5.02 (m, 2H), 5.04 (d, *J* = 1.6 Hz, 1H), 5.90 (dt, *J* = 15.6, 6.8 Hz, 1H), 6.15 (d, *J* = 15.2 Hz, 1H), 7.14–7.32 (m, 10H); ^{13}C NMR: δ = 29.9, 35.0, 35.5, 39.1, 113.2, 113.5, 125.6, 126.0, 128.2, 128.4, 128.6, 131.3, 131.7, 140.2, 142.4, 147.8, 148.0; HRMS (EI^+): Calcd for $C_{22}H_{24}$, M^+ 288.1878. Found *m/z* 288.1872.

3d: IR (neat): 2922, 2851, 1448 cm^{-1}; ^{1}H NMR: δ = 0.80–0.94 (m, 2H), 1.04–1.36 (m, 11H), 1.60–1.78 (m, 10H), 1.96–2.08 (m, 1H), 2.21 (t, *J* = 7.2 Hz, 2H), 4.90 (d, *J* = 2.4 Hz, 1H), 4.95 (s, 2H), 4.99 (d, *J* = 1.6 Hz, 1H), 5.69 (dd, *J* = 15.6, 7.2 Hz, 1H), 6.02 (d, *J* = 15.6 Hz, 1H); ^{13}C NMR: δ = 26.0, 26.2, 26.4, 26.7, 32.6, 32.8, 33.3, 36.0, 37.4, 40.8, 112.1, 112.6, 127.7, 139.0, 148.6, 149.2; HRMS (EI^+): Calcd for $C_{20}H_{32}$, M^+ 272.2504. Found *m/z* 272.2498.

3e: IR (neat): 2925, 2854, 1454, 1362, 1074 cm^{-1}; ^{1}H NMR: δ = 1.75 (quint, *J* = 6.8 Hz, 2H), 2.33 (t, *J* = 7.6 Hz, 2H), 3.48 (t, *J* = 6.8 Hz, 2H), 4.09 (d, *J* = 6.0 Hz, 2H), 4.50 (s, 2H), 4.53 (s, 2H), 4.99 (s, 1H), 5.02 (s, 2H), 5.12 (s, 1H), 5.87 (dt, *J* = 15.6, 6.8 Hz, 1H), 6.33 (d, *J* = 16.0 Hz, 1H), 7.22–7.40 (m, 10H); ^{13}C NMR: δ = 28.2, 31.8, 69.8, 70.5, 72.1, 72.9, 113.8, 114.5, 127.5, 127.6, 127.8, 128.3, 128.4, 132.8, 138.2, 138.6, 147.1, 147.3; HRMS (FAB^+): Calcd for $C_{24}H_{28}O_2Na$, $M+Na^+$ 371.1987. Found *m/z* 371.1992.

3f: IR (neat): 2935, 2855, 1453, 1103 cm^{-1}; ^{1}H NMR: δ = 1.34–1.48 (m, 4H), 1.63 (quint, *J* = 7.2 Hz, 2H), 1.74 (quint, *J* = 7.2 Hz, 2H), 2.16–2.26 (m, 4H), 3.47 (t, *J* = 6.4 Hz, 2H), 3.49 (t, *J* = 6.4 Hz, 2H), 4.51 (s, 2H), 4.51 (s, 2 H), 4.91 (s, 1H), 4.95 (s, 2H), 5.00 (s, 1H), 5.74 (dt, *J* = 15.6, 6.8 Hz, 1H), 6.08 (d, *J* = 16.0 Hz, 1H), 7.24–7.40 (m, 10H); ^{13}C NMR: δ = 25.9, 28.0, 29.26, 29.31, 29.6, 35.2, 69.6, 70.4, 72.8, 72.9, 112.5, 113.0, 127.4, 127.5, 127.6, 128.31, 128.33, 130.8, 132.3, 138.5, 138.6, 148.1, 148.5; HRMS (ESI^+): Calcd for $C_{28}H_{36}O_2Na$, $M+Na^+$ 427.2613. Found *m/z* 427.2584.

3g: IR (neat): 2930, 1472, 1256, 1104 cm^{-1}; ^{1}H NMR: δ = 0.05 (s, 12H), 0.89 (s, 18H), 1.20–1.70 (m, 8H), 2.10–2.30 (m, 4H), 3.52–3.69 (m, 4H), 4.90 (s, 1H), 4.95 (s, 2H), 4.98 (s, 1H), 5.73 (dt, *J* = 15.6, 6.8 Hz, 1H), 6.08 (d, *J* = 15.6 Hz, 1H); ^{13}C NMR: δ = −5.30, −5.27, 18.3, 18.4, 25.5, 25.96, 25.97, 28.0, 29.0, 32.3, 32.7, 35.4, 62.5, 63.2, 112.4, 113.0, 130.6, 132.6, 148.2, 148.6; HRMS (EI^+): Calcd for $C_{26}H_{52}O_2Si_2$, M^+ 452.3506. Found *m/z* 452.3504.

3h: IR (neat): 3300, 2934, 2860, 1435 cm^{-1}; ^{1}H NMR: δ = 1.30–1.74 (m, 8H), 2.14–2.26 (m, 4H), 3.58–3.70 (m, 4H), 4.91 (d, *J* = 2.0 Hz, 1H), 4.95 (s, 1H), 4.96 (s, 1H), 4.99 (s, 1H), 5.75 (dt, *J* = 15.6, 7.2 Hz, 1H), 6.10 (d, *J* = 15.6 Hz, 1H); ^{13}C NMR: δ = 25.3, 27.8, 29.0, 32.0, 32.5, 35.3, 62.3, 62.8, 112.7, 113.2, 130.9, 132.2, 148.0, 148.3; HRMS (FAB^+): Calcd for $C_{14}H_{24}O_2Na$, $M+Na^+$ 247.1674. Found *m/z* 247.1669.

3i: IR (neat): 2936, 2863, 2245, 1585, 1424 cm^{-1}; ^{1}H NMR: δ = 1.38–1.50 (m, 4H), 1.66 (quint, *J* = 6.8 Hz, 2H), 1.78 (quint, *J* = 7.2 Hz, 2H), 2.18–2.40 (m, 8H), 4.95 (s, 1H), 4.97 (s, 1H), 4.98 (s, 1H), 5.04 (s, 1H), 5.65 (dt, *J* = 15.2, 7.2 Hz, 1H), 6.14 (d, *J* = 15.6 Hz, 1H); ^{13}C NMR: δ = 16.5, 17.1, 24.8, 25.2, 27.2, 28.2, 31.4, 34.9, 113.5, 113.8, 119.5, 119.7, 129.9, 132.4, 147.4, 147.6; HRMS (EI^+): Calcd for $C_{16}H_{21}N_2$, $M-H^+$ 241.1699. Found *m/z* 241.1694.

3j: IR (neat): 2921, 2851, 1448 cm^{-1}; ^{1}H NMR: δ = 0.80–0.94 (m, 2H), 1.06–1.76 (m, 15H), 2.10–2.26 (m, 6H), 4.86 (d, *J* = 0.8 Hz, 1H), 4.88 (d, *J* = 0.8 Hz, 1H), 5.09 (d, *J* = 2.4 Hz, 1H), 5.20 (d, *J* = 1.6 Hz, 1H), 5.71 (s, 1H); ^{13}C NMR: δ = 26.4, 26.6, 26.8, 28.1, 28.8, 29.7, 33.5, 36.2, 37.3, 42.1, 113.5, 114.5, 121.8, 143.0, 144.9, 146.8; HRMS (EI^+): Calcd for $C_{18}H_{28}$, M^+ 244.2191. Found *m/z* 244.2188.

Procedure for Diels–Alder reaction of *N*-phenyl-1,2,4-triazoline-3,5-dione (4) with 3a.
To a side-arm tube equipped with a stirrer bar, was added *N*-phenyl-1,2,4-triazoline-3,5-dione (**4**, 62.1 mg, 0.36 mmol, 2.4 equiv). The tube was evacuated and refilled with argon three times. Then, toluene (1.5 mL) and **3a** (46.6 mg, 0.15 mmol) were added via syringe, and the tube was closed. After stirring at 0 °C for 1 h, the solvent was removed by evaporation and the residue purified by preparative thin-layer chromatography (hexane:ethyl acetate = 3:1) to give the two isomeric bisadducts (**5a**, 74.3 mg, 0.11 mmol, 75% major) and (**5a'**, 5.3 mg, 0.01 mmol, 6% minor).

5a: IR (KBr): 2926, 2855, 1772, 1718, 1503, 1419 cm^{-1}; ^{1}H NMR: δ = 0.80–0.92 (m, 6H), 1.16–1.52 (m, 24H), 1.88–1.99 (m, 1H), 2.16–2.40 (m, 3H), 3.98 (d, *J* = 13.6 Hz, 1H), 4.06–4.20 (m, 2H), 4.52–4.58 (m, 1H), 4.83–4.93 (m, 1H), 4.94 (d, *J* = 13.6 Hz, 1H) 7.34–7.56 (m, 10H); ^{13}C NMR: δ = 14.0, 14.1, 22.56, 22.61, 25.3, 28.7, 29.1, 29.2, 29.3, 29.38, 29.40, 30.9, 31.7, 31.8, 33.4, 44.8, 46.2, 57.4, 58.5, 119.3, 125.3, 125.4, 128.1, 128.5, 129.1, 129.3, 130.7, 131.4, 132.6, 149.4, 152.5, 152.7, 153.2; HRMS (ESI^{+}): Calcd for $C_{38}H_{51}N_{6}O_{4}$, $M+H^{+}$ 655.3972. Found *m/z* 655.3975.

5a': IR (KBr): 2923, 2855, 1773, 1719, 1600, 1502, 1413 cm^{-1}; ^{1}H NMR: δ = 0.80–0.94 (m, 6H), 1.14–1.46 (m, 24H), 1.74–1.94 (m, 2H), 2.04–2.16 (m, 1H), 2.38–2.48 (m, 1H), 3.39 (d, *J* = 12.0 Hz, 1H), 3.96 (d, *J* = 12.8 Hz, 1H), 4.56 (d, *J* = 13.2 Hz, 1H), 4.58–4.66 (m, 1H), 5.14 (d, *J* = 12.0 Hz, 1H), 6.24 (d, *J* = 4.4 Hz, 1H), 7.34–7.56 (m, 10H); ^{13}C NMR: δ = 14.07, 14.09, 22.56, 22.64, 23.6, 25.0, 29.0, 29.19, 29.25, 29.4, 29.5, 31.0, 31.7, 31.8, 31.9, 47.2, 52.4, 52.5, 61.8, 125.41, 125.43, 126.1, 128.0, 128.37, 128.44, 129.2, 130.8, 131.0, 150.2, 151.4, 151.8, 152.0; HRMS (EI^{+}): Calcd for $C_{38}H_{50}N_{6}O_{4}$, M^{+} 654.3894. Found *m/z* 654.3923.

Procedure for Diels–Alder reaction of tetracyanoethylene (6) with 3a.
To a side-arm tube equipped with a stirrer bar, was added tetracyanoethylene (**6**, 51.5 mg, 0.40 mmol, 2.7 equiv). The tube was evacuated and refilled with argon three times. Then, toluene (1.5 mL) and **3a** (46.1 mg, 0.15 mmol) were added via syringe and the tube was closed. After stirring at 60 °C for 24 h, the solvent was removed by evaporation and the residue purified by preparative thin-layer chromatography (hexane:ethyl acetate = 3:1) to give an isomeric mixture of **7a** and **7a'** (52.7 mg, 0.12 mmol, 62% major, 19% minor).

7a: IR (neat): 2927, 2855, 1611, 1467 cm^{-1}; ^{1}H NMR: δ = 0.82–0.96 (m, 6H), 1.20–1.78 (m, 25H), 1.92–2.02 (m, 1H), 2.24 (t, *J* = 7.6 Hz, 2H) 3.07-3.14 (m, 1H), 3.18 (d, *J* = 17.6 Hz, 1H), 3.29 (d, *J* = 17.2 Hz, 1H), 5.08 (s, 1H), 5.15 (s, 1H), 5.79–5.83 (m, 1H); ^{13}C NMR: δ = 22.8, 22.9, 26.8, 28.4, 29.1, 29.4, 29.5, 29.57, 29.61, 29.7, 31.8, 32.1, 32.2, 33.5, 33.7, 39.3, 41.9, 43.9, 109.1, 110.6, 111.4, 111.6, 114.4, 120.7, 131.2, 144.9; HRMS (EI^{+}): Calcd for $C_{28}H_{40}N_{4}$, M^{+} 432.3253. Found *m/z* 432.3249.

References
1) Giordano, G.; Crabtree, R. H. *Inorg. Synth.* 1979, *19*, 218.
2) Brandsma, L. *Synthesis of Acetylenes, Allenes and Cumulenes: Methods and Techniques*; Elsevier: 2004; p 243.
3) Trost, B. M.; Pinkerton, A. B.; Seidel, M. *J. Am. Chem. Soc.* 2001, *123*, 12466.
4) Zhang, Z.; Liu, C.; Kinder, R. E.; Han, X.; Qian, H.; Widenhoefer, R. A. *J. Am. Chem. Soc.* 2006, *128*, 9066.

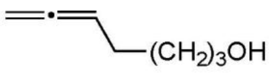

1h

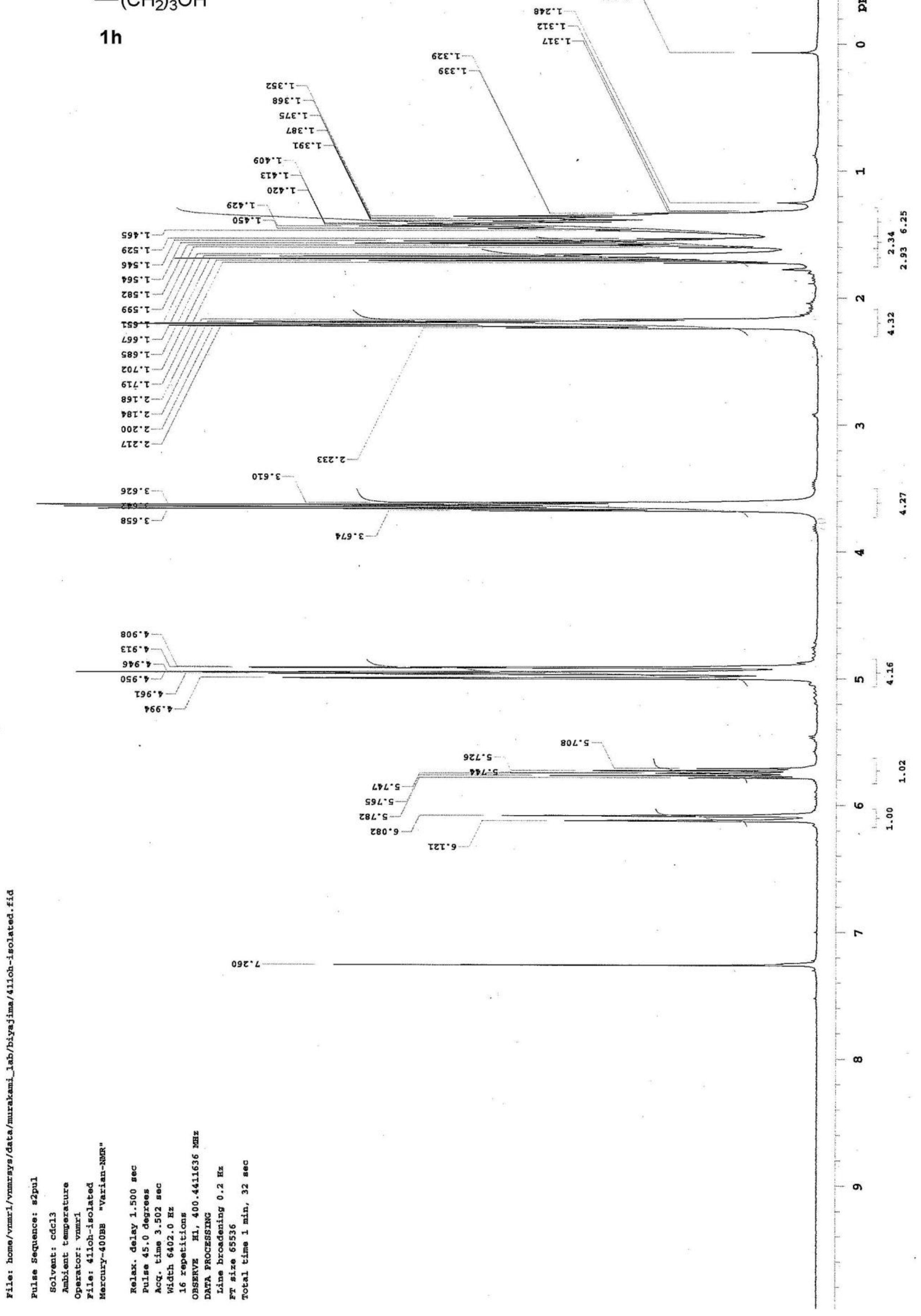
File: home/vnmr1/vnmrsys/data/murakami_lab/biyajima/411oh-isolated.fid
Pulse Sequence: s2pul
Solvent: cdcl3
Ambient temperature
Operator: vnmr1
File: 411oh-isolated
Mercury-400BB "Varian-NMR"
Relax. delay 1.500 sec
Pulse 45.0 degrees
Acq. time 3.502 sec
Width 6402.0 Hz
16 repetitions
OBSERVE H1, 400.4411636 MHz
DATA PROCESSING
Line broadening 0.2 Hz
FT size 65536
Total time 1 min, 32 sec
ppm

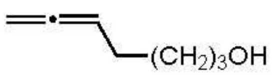
(CH2)3OH

1h

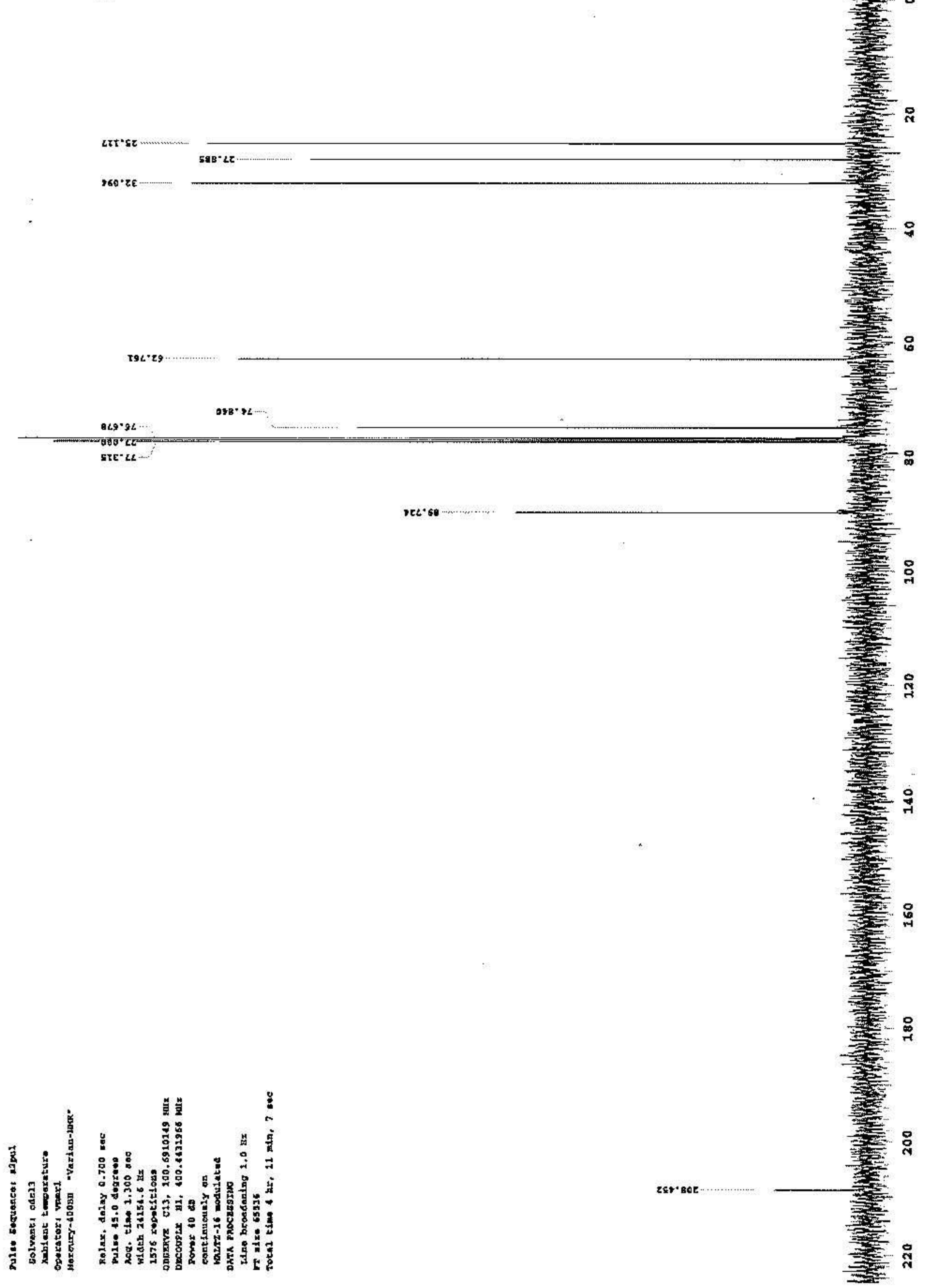
25.117
27.885
32.094
62.761
74.840
76.678
77.000
77.315
89.724
208.452
ppm

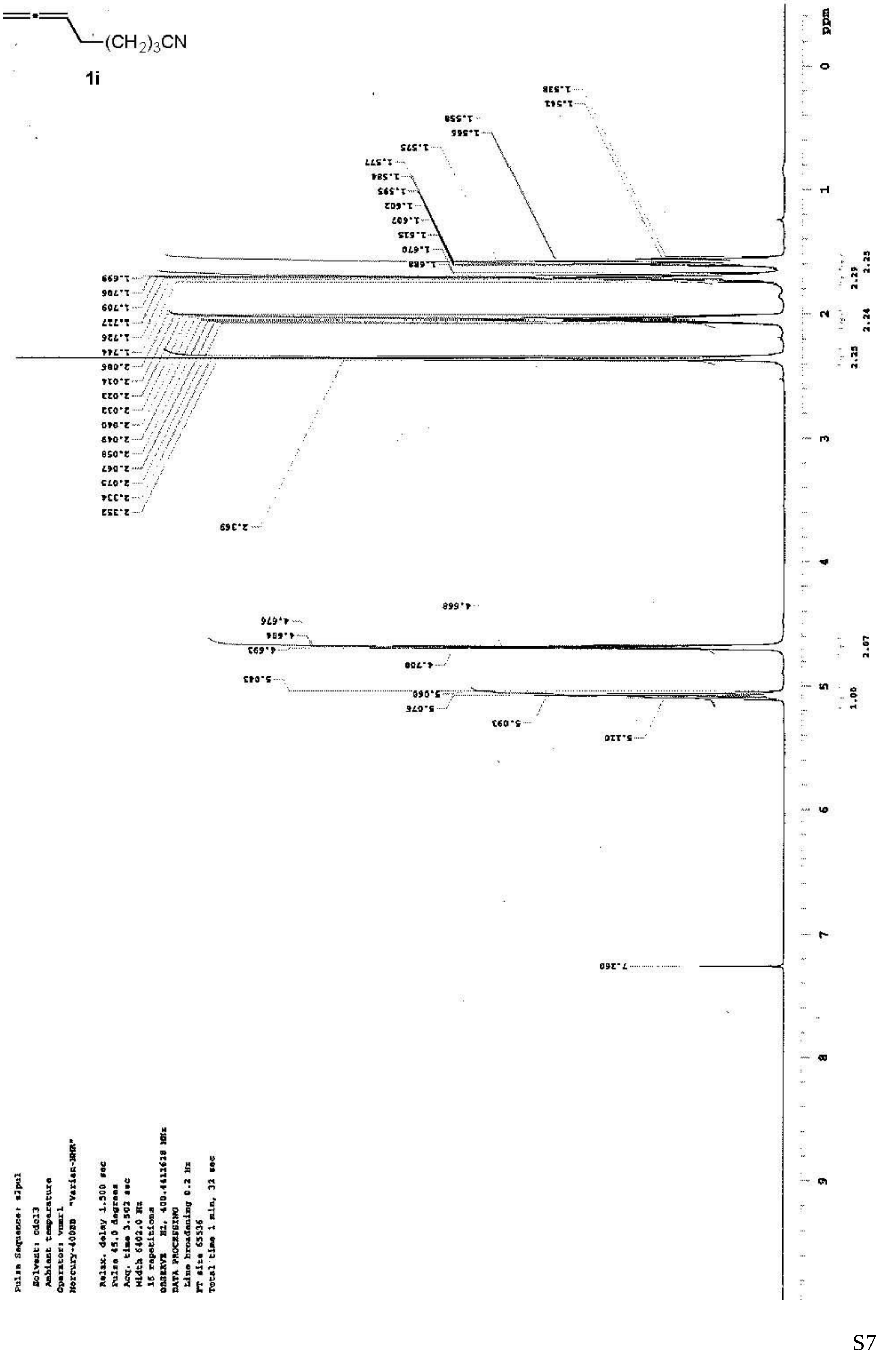

1i

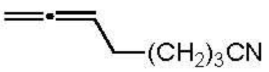
(CH2)3CN

1i

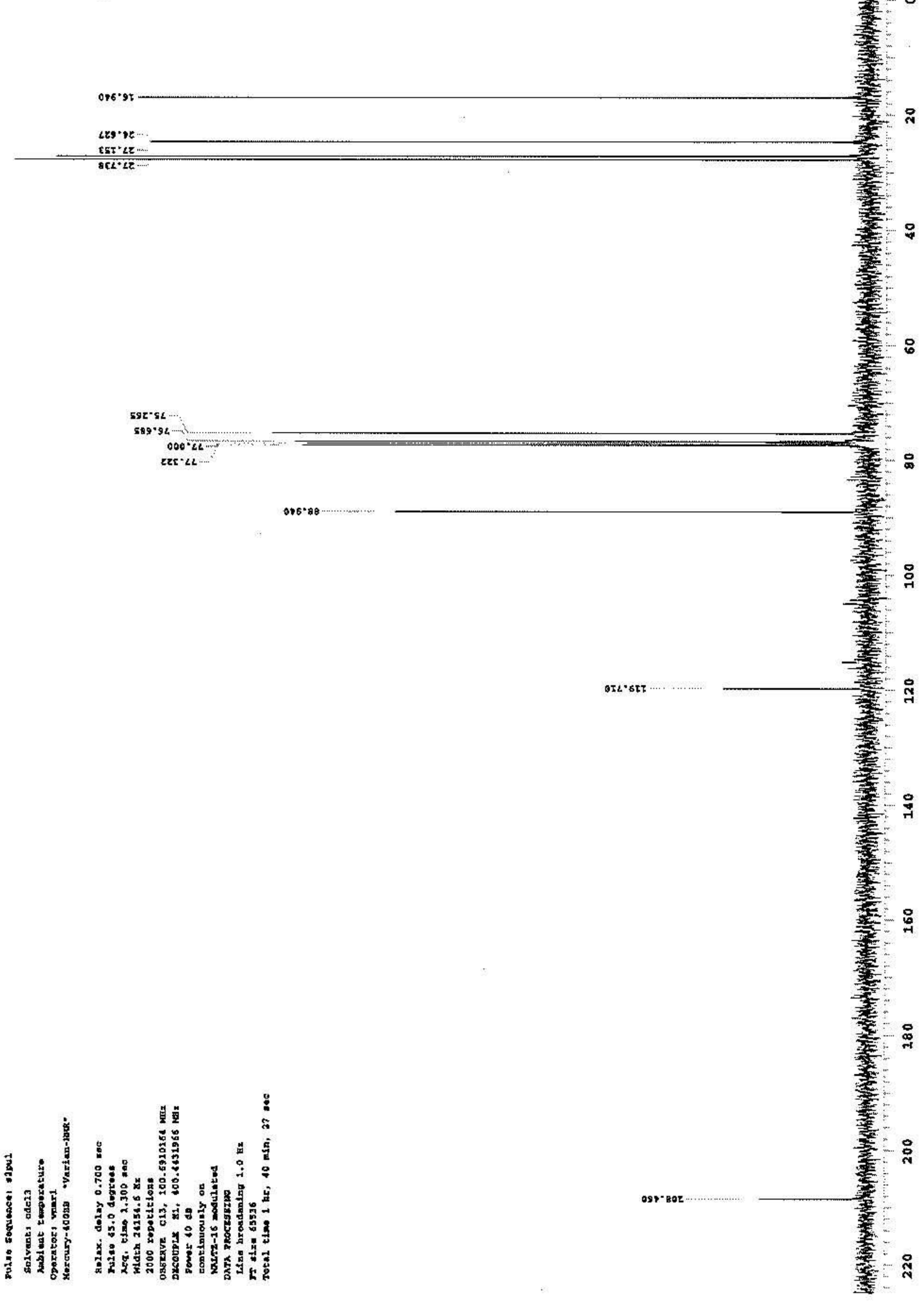
16.940
24.627
27.153
27.738
75.365
76.685
77.000
77.322
88.940
119.710
208.460
ppm
0
20
40
60
80
100
120
140
160
180
200
220

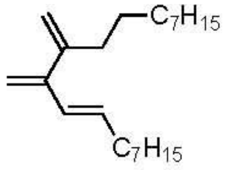

3a

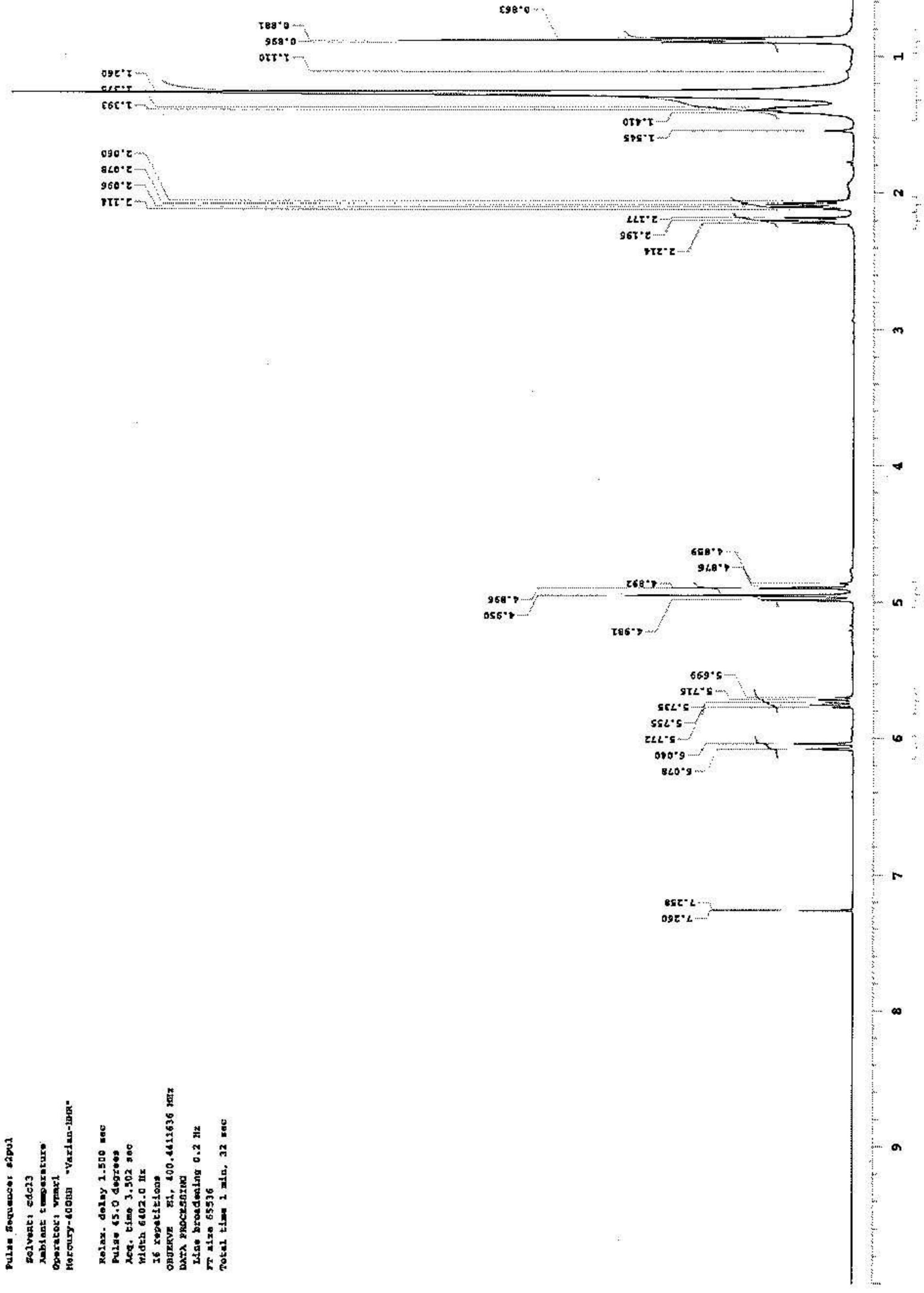

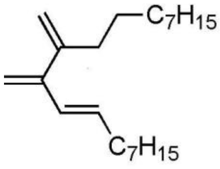
C7H15
C7H15

3a

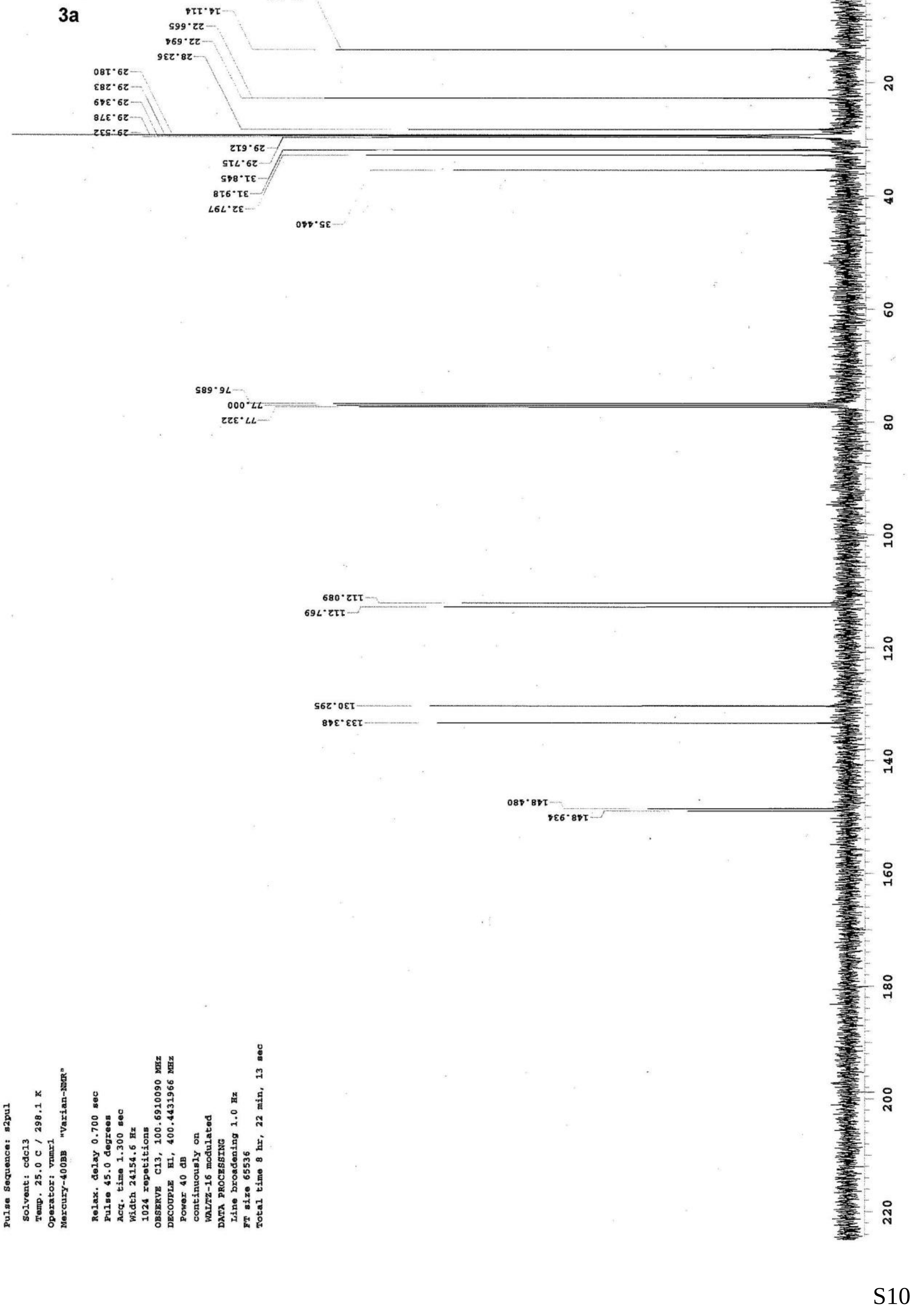
Pulse Sequence: s2pul
Solvent: cdcl3
Temp. 25.0 C / 298.1 K
Operator: vnmr1
Mercury-400BB "Varian-NMR"
Relax. delay 0.700 sec
Pulse 45.0 degrees
Acq. time 1.300 sec
Width 24154.6 Hz
1024 repetitions
OBSERVE C13, 100.6910090 MHz
DECOUPLE H1, 400.4431966 MHz
Power 40 dB
continuously on
WALTZ-16 modulated
DATA PROCESSING
Line broadening 1.0 Hz
FT size 65536
Total time 8 hr, 22 min, 13 sec
14.092
14.114
22.665
22.694
28.236
29.180
29.283
29.349
29.378
29.532
29.612
29.715
31.845
31.918
32.797
35.440
76.685
77.000
77.322
112.089
112.769
130.295
133.348
148.480
148.934
ppm
0
20
40
60
80
100
120
140
160
180
200
220

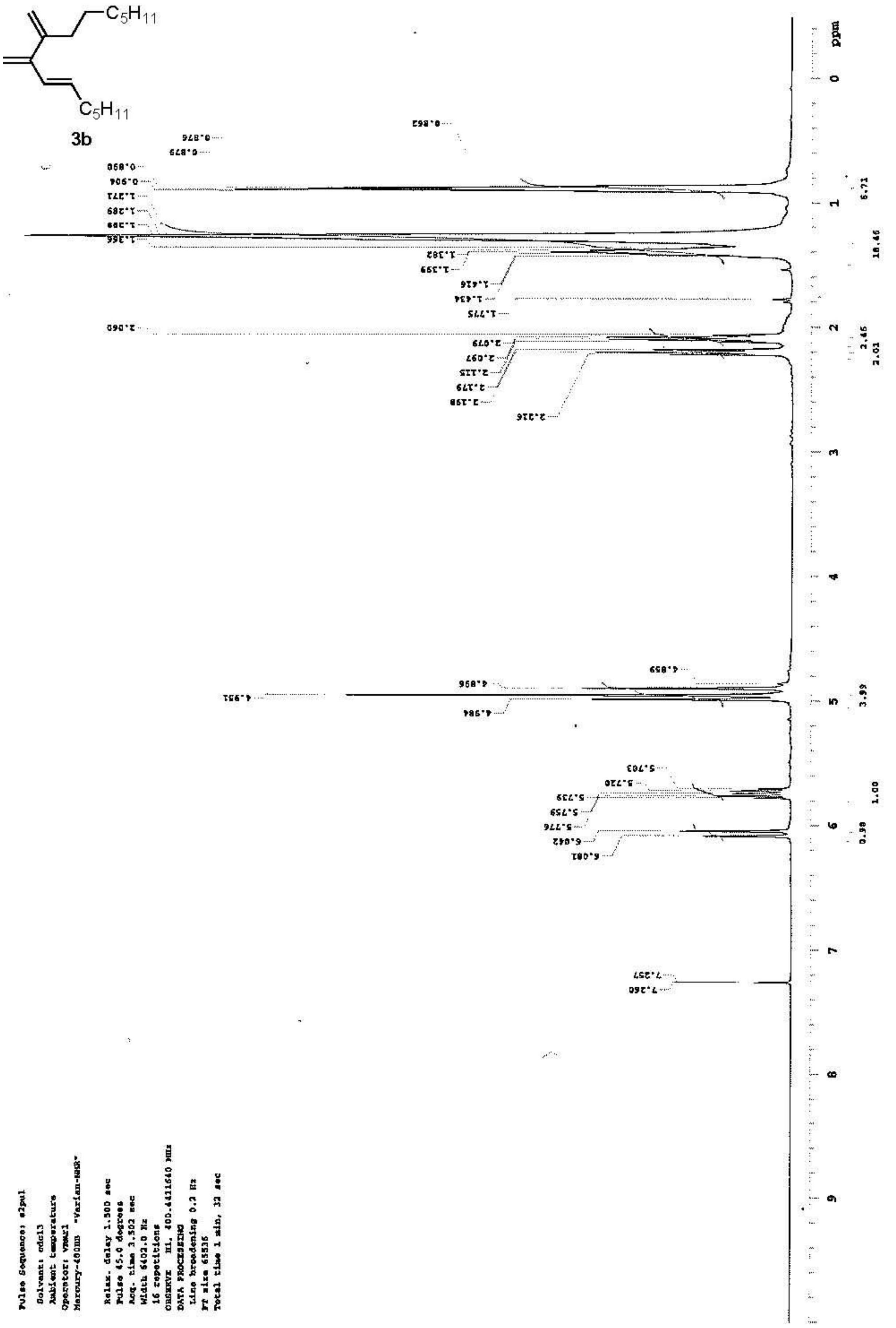
C5H11
C5H11
3b
Pulse Sequence: s2pul
Solvent: cdcl3
Ambient temperature
Operator: vnmr1
Mercury-400BB "Varian-NMR"
Relax. delay 1.500 sec
Pulse 45.0 degrees
Acq. time 3.502 sec
Width 6402.0 Hz
16 repetitions
OBSERVE H1, 400.4411640 MHz
DATA PROCESSING
Line broadening 0.2 Hz
FT size 65536
Total time 1 min, 32 sec
ppm

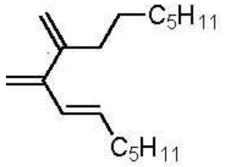
C5H11
C5H11

3b

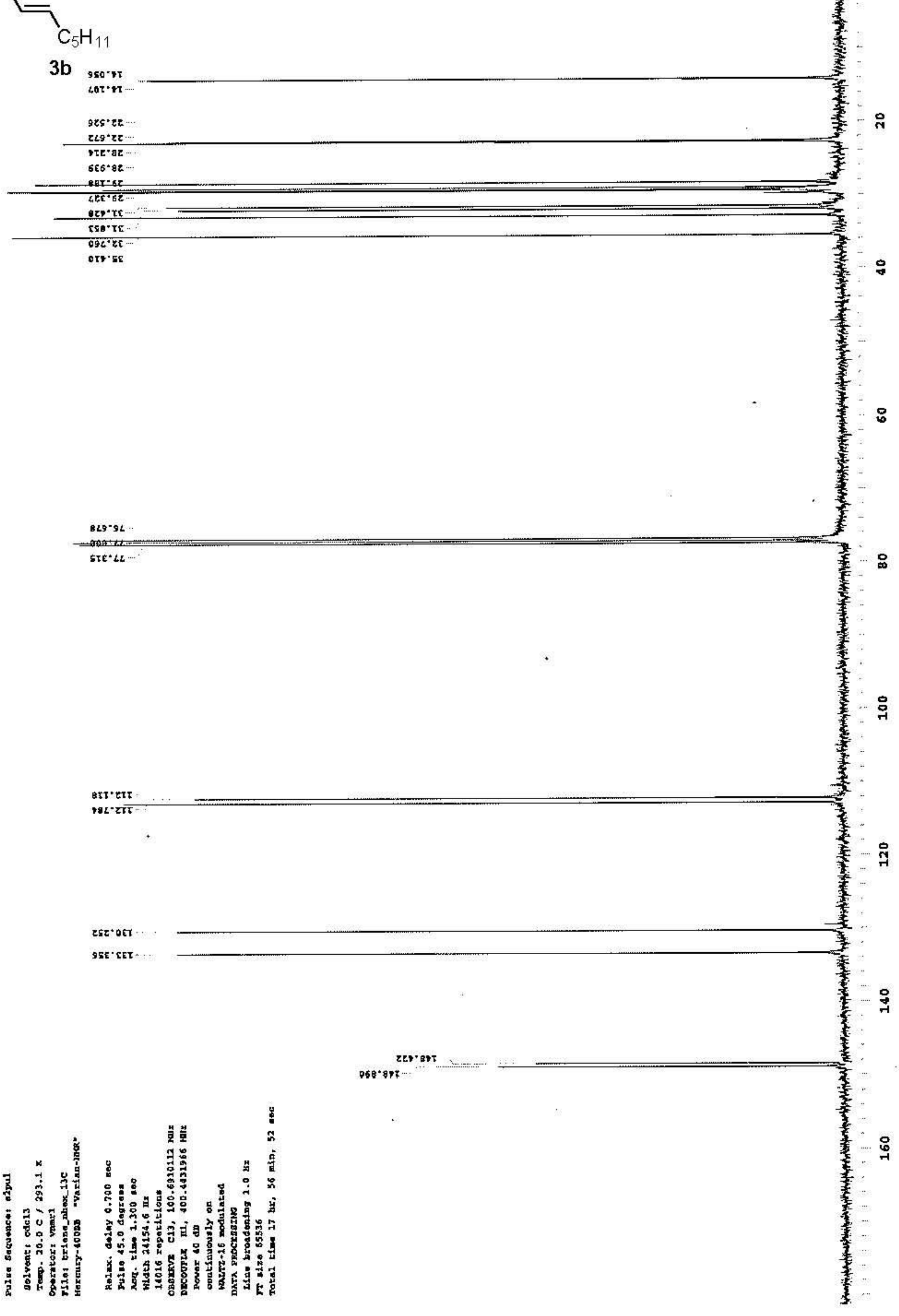
Pulse Sequence: s2pul
Solvent: cdcl3
Temp. 20.0 C / 293.1 K
Operator: vnmr1
File: triene_nhex_13C
Mercury-400BB "Varian-NMR"
Relax. delay 0.700 sec
Pulse 45.0 degrees
Acq. time 1.300 sec
Width 24154.6 Hz
14016 repetitions
OBSERVE C13, 100.6910112 MHz
DECOUPLE H1, 400.4431966 MHz
Power 40 dB
continuously on
WALTZ-16 modulated
DATA PROCESSING
Line broadening 1.0 Hz
FT size 65536
Total time 17 hr, 56 min, 52 sec
148.890
148.422
133.356
130.252
112.784
112.118
77.315
77.000
76.678
35.410
32.760
31.853
31.428
29.327
29.188
28.939
28.214
22.672
22.526
14.107
14.056
160
140
120
100
80
60
40
20
ppm

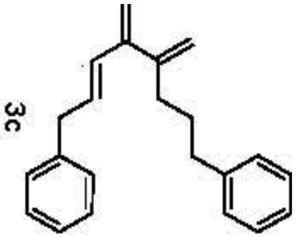

3c

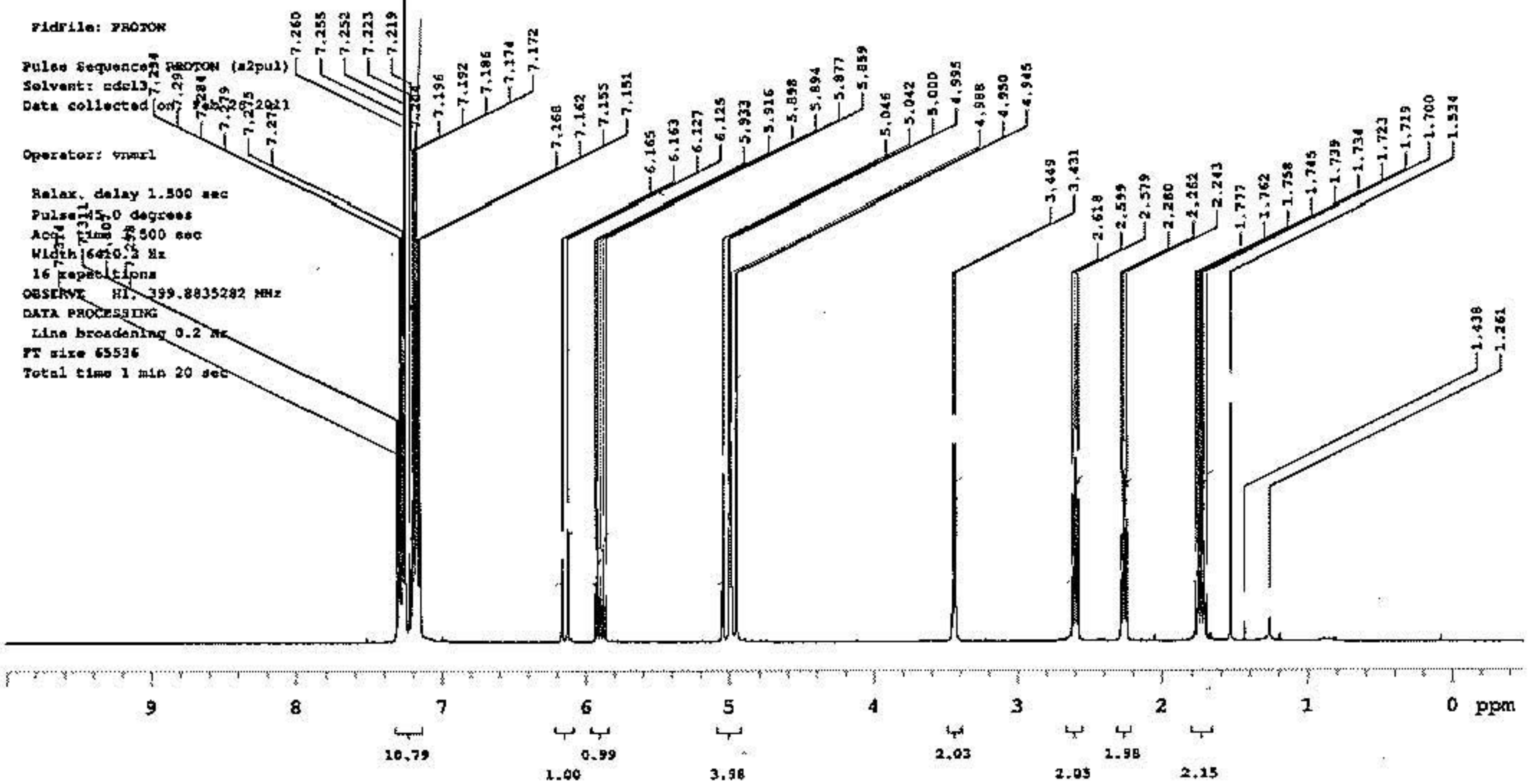
Sample Name:
Data Collected on:
400-HR-vnmrs400
Archive directory:
Sample directory:
FidFile: PROTON
Pulse Sequence: PROTON (s2pul)
Solvent: cdcl3
Data collected on: Feb 2 2021
Operator: vnmrl
Relax. delay 1.500 sec
Pulse 45.0 degrees
Acq. time 2.500 sec
Width 6410.3 Hz
16 repetitions
OBSERVE H1, 399.8835282 MHz
DATA PROCESSING
Line broadening 0.2 Hz
FT size 65536
Total time 1 min 20 sec
7.260
7.255
7.252
7.223
7.219
7.204
7.196
7.192
7.186
7.174
7.172
7.168
7.162
7.155
7.151
6.165
6.163
6.127
6.125
5.933
5.916
5.898
5.894
5.877
5.859
5.046
5.042
5.000
4.995
4.988
4.950
4.945
3.449
3.431
2.618
2.599
2.579
2.280
2.262
2.243
1.777
1.762
1.758
1.745
1.739
1.734
1.723
1.719
1.700
1.534
1.438
1.261
9
8
7
6
5
4
3
2
1
0 ppm
10.79
1.00
0.99
3.98
2.03
2.05
1.98
2.15

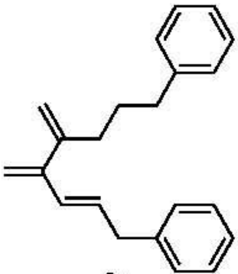

3c

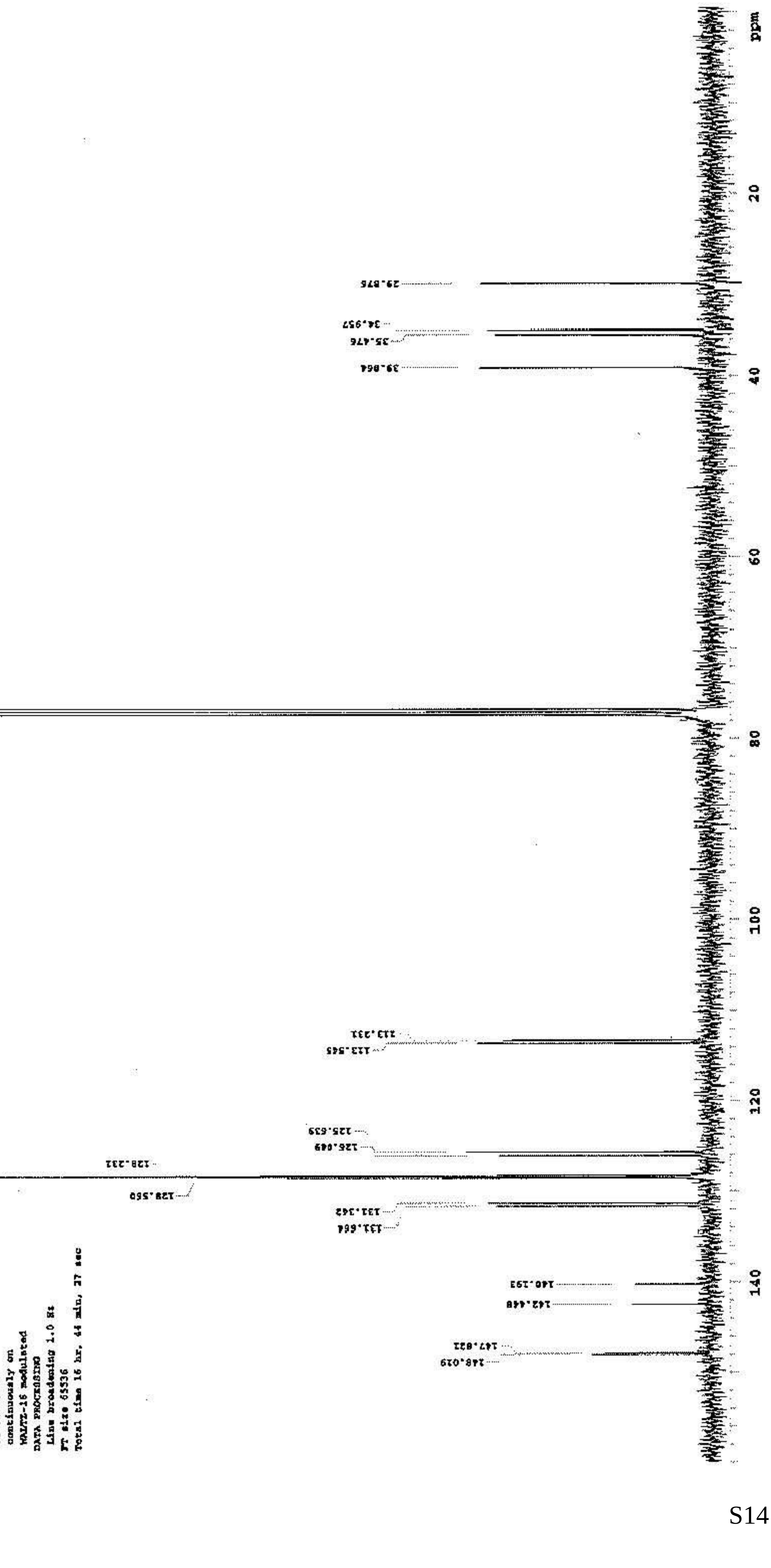

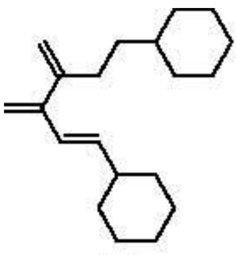

3d

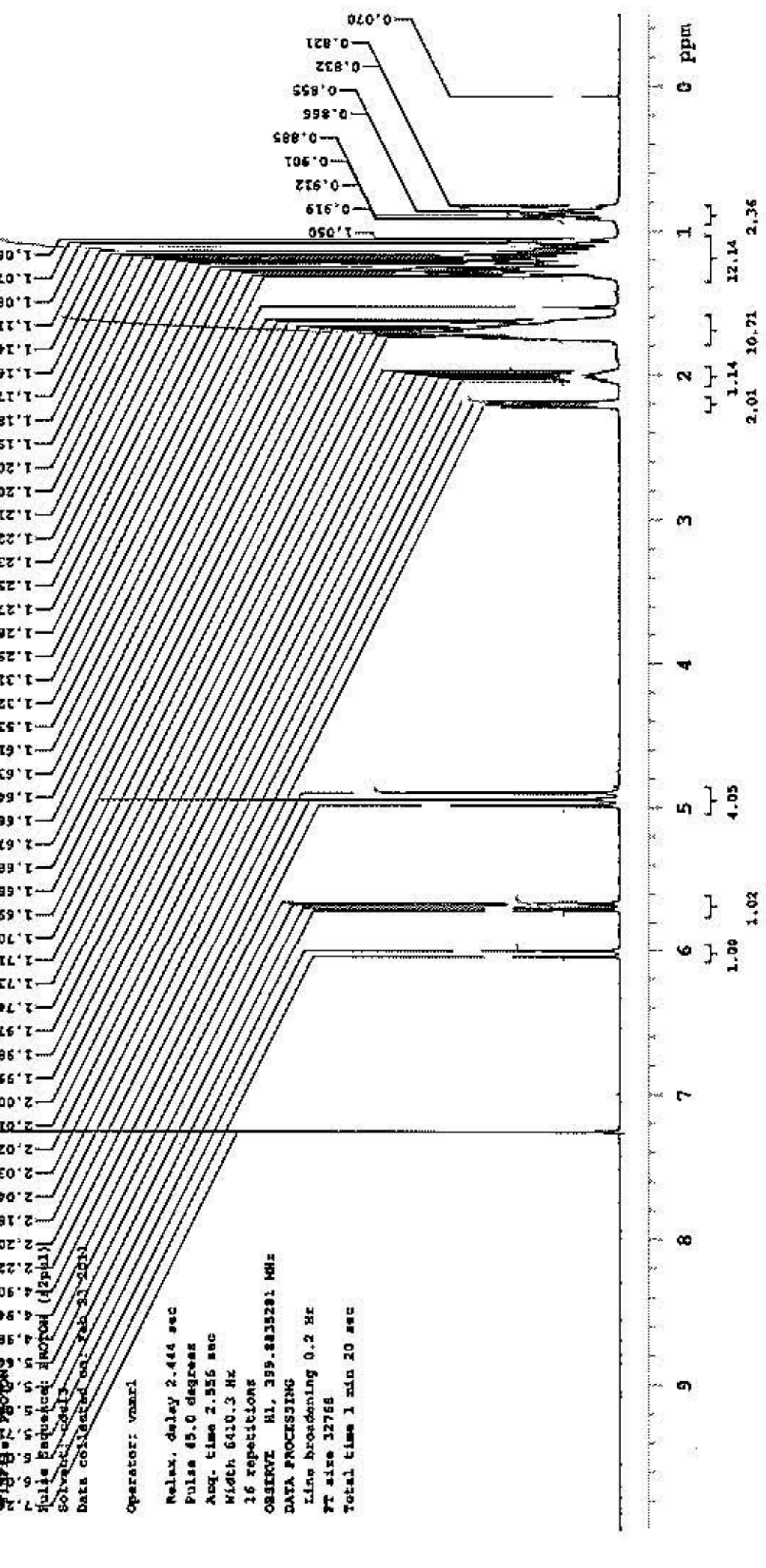

Sample Name:
Data Collected on:
400-MR-vnmrs400
Archive directory:
Sample directory:
Pulse Sequence: PROTON (s2pul)
Solvent: cdcl3
Operator: vnmr1
Relax. delay 2.444 sec
Pulse 45.0 degrees
Acq. time 2.556 sec
Width 6410.3 Hz
16 repetitions
OBSERVE H1, 399.8835281 MHz
DATA PROCESSING
Line broadening 0.2 Hz
FT size 32768
Total time 1 min 20 sec
ppm
2.36
12.14
10.71
1.14
2.01
4.05
1.02
1.00

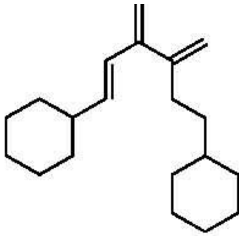

3d

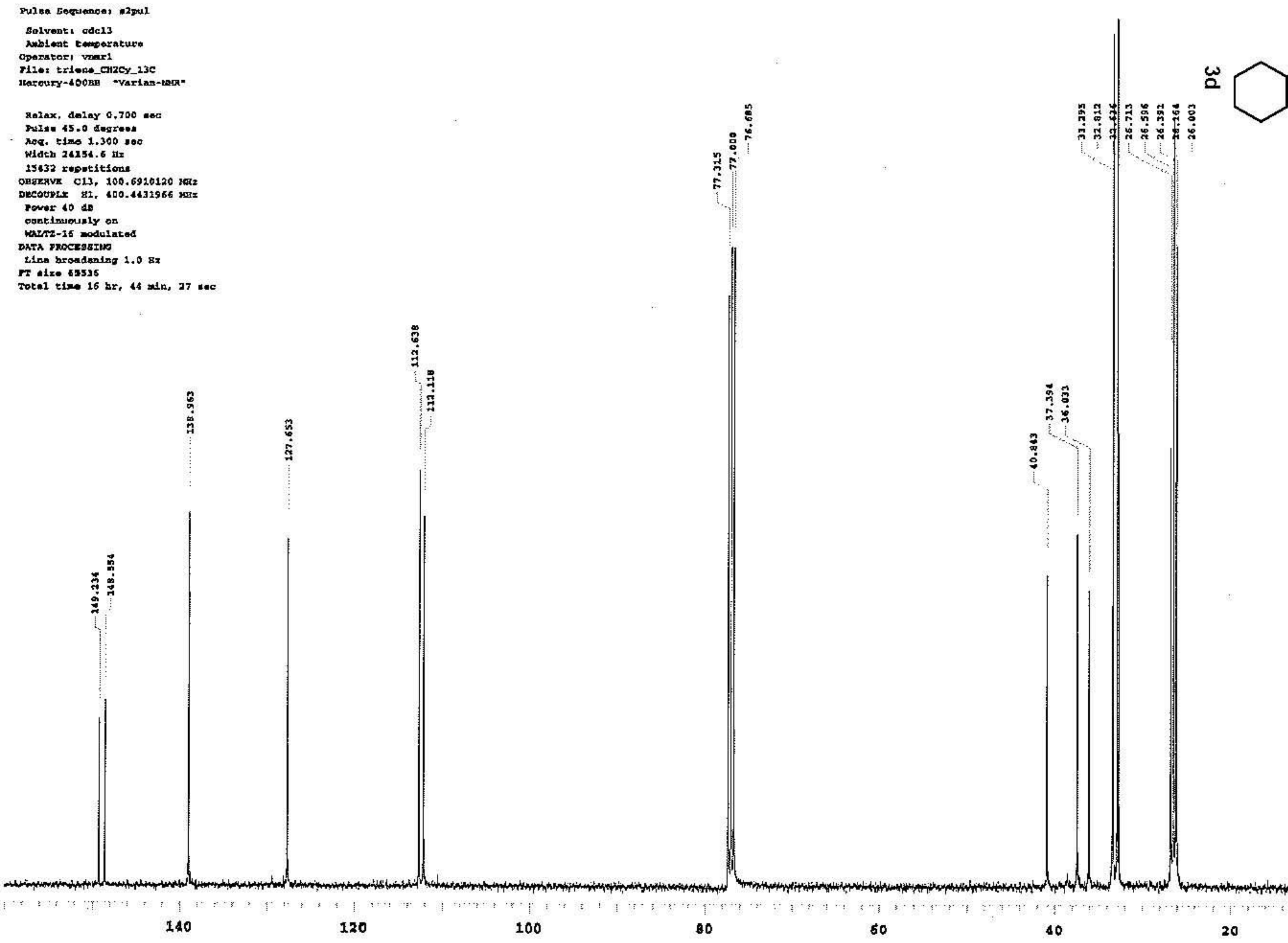

Pulse Sequence: s2pul
Solvent: cdcl3
Ambient temperature
Operator: vnmr1
File: triene_CH2Cy_13C
Mercury-400BB "Varian-NMR"
Relax. delay 0.700 sec
Pulse 45.0 degrees
Acq. time 1.300 sec
Width 24154.6 Hz
15432 repetitions
OBSERVE C13, 100.6910120 MHz
DECOUPLE H1, 400.4431966 MHz
Power 40 dB
continuously on
WALTZ-16 modulated
DATA PROCESSING
Line broadening 1.0 Hz
FT size 65536
Total time 16 hr, 44 min, 27 sec
149.234
148.554
138.963
127.653
112.638
112.118
77.315
77.000
76.685
40.843
37.394
36.031
33.295
32.812
26.713
26.596
26.392
26.003
140
120
100
80
60
40
20
ppm

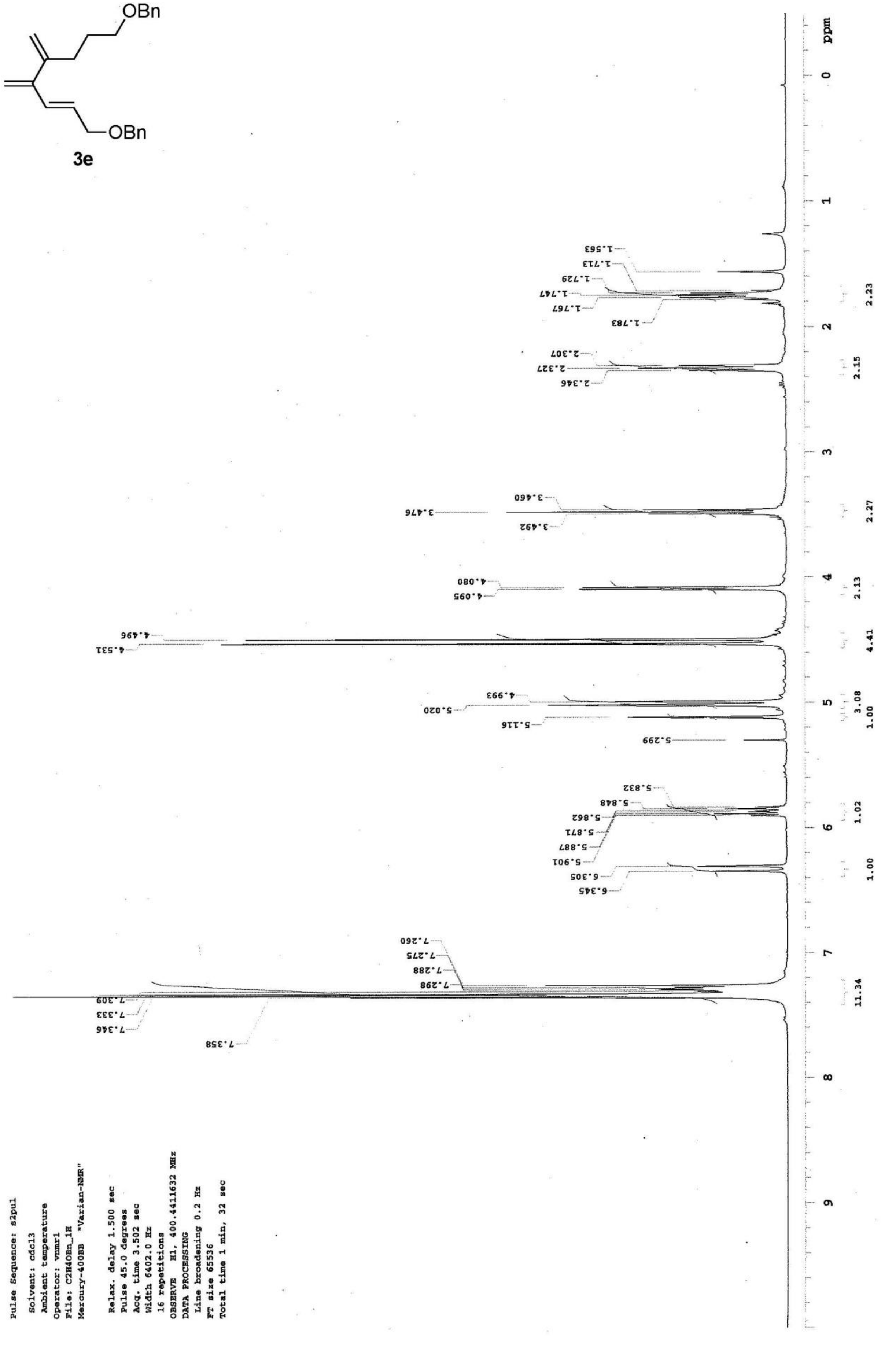
OBn
OBn
3e
Pulse Sequence: s2pul
Solvent: cdcl3
Ambient temperature
Operator: vnmr1
File: C2H4OBn_1H
Mercury-400BB "Varian-NMR"
Relax. delay 1.500 sec
Pulse 45.0 degrees
Acq. time 3.502 sec
Width 6402.0 Hz
16 repetitions
OBSERVE H1, 400.4411632 MHz
DATA PROCESSING
Line broadening 0.2 Hz
FT size 65536
Total time 1 min, 32 sec
ppm

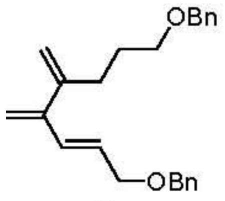
OBn
OBn

3e

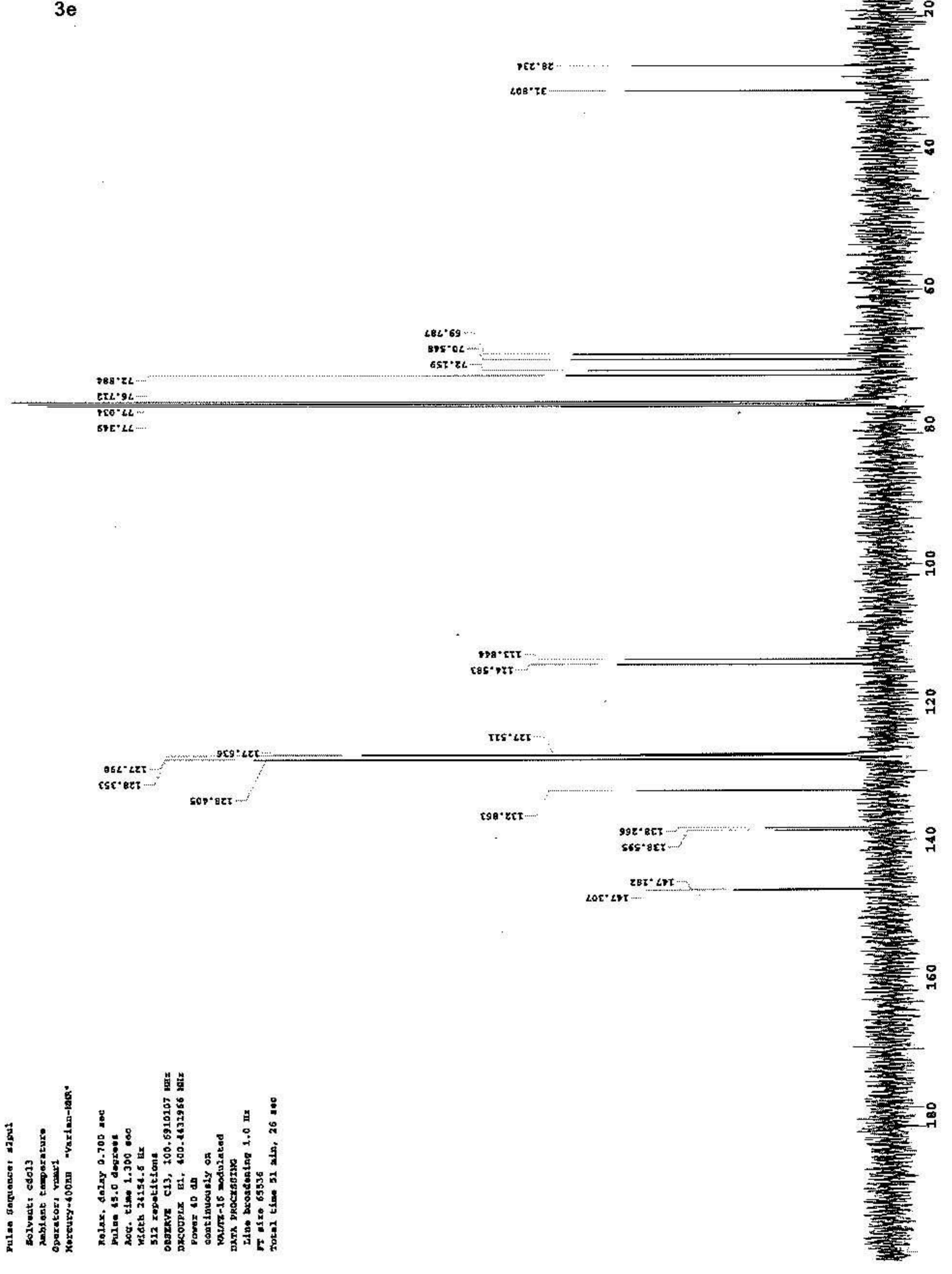
Pulse Sequence: s2pul
Solvent: cdcl3
Ambient temperature
Operator: vnmr1
Mercury-400BB "Varian-NMR"
Relax. delay 0.700 sec
Pulse 45.0 degrees
Acq. time 1.300 sec
Width 24154.6 Hz
512 repetitions
OBSERVE C13, 100.5910107 MHz
DECOUPLE H1, 400.4431956 MHz
Power 40 dB
continuously on
WALTZ-16 modulated
DATA PROCESSING
Line broadening 1.0 Hz
FT size 65536
Total time 51 min, 26 sec
28.234
31.807
69.787
70.548
72.159
72.884
76.713
77.034
77.349
113.844
114.583
127.511
127.636
127.790
128.353
128.405
132.863
138.266
138.595
147.182
147.207
ppm
20
40
60
80
100
120
140
160
180

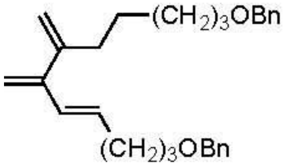

3f

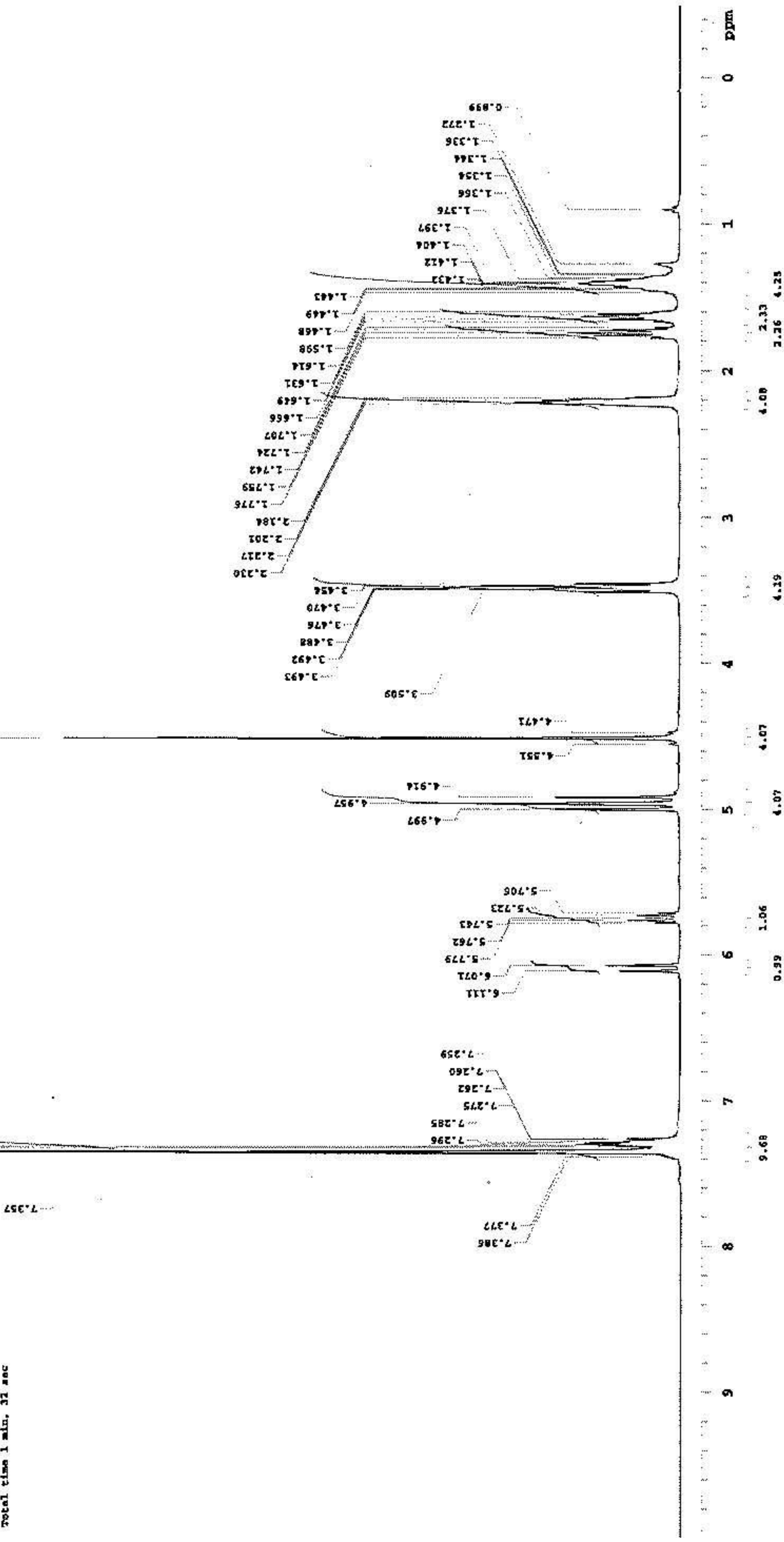

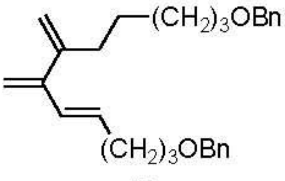

3f

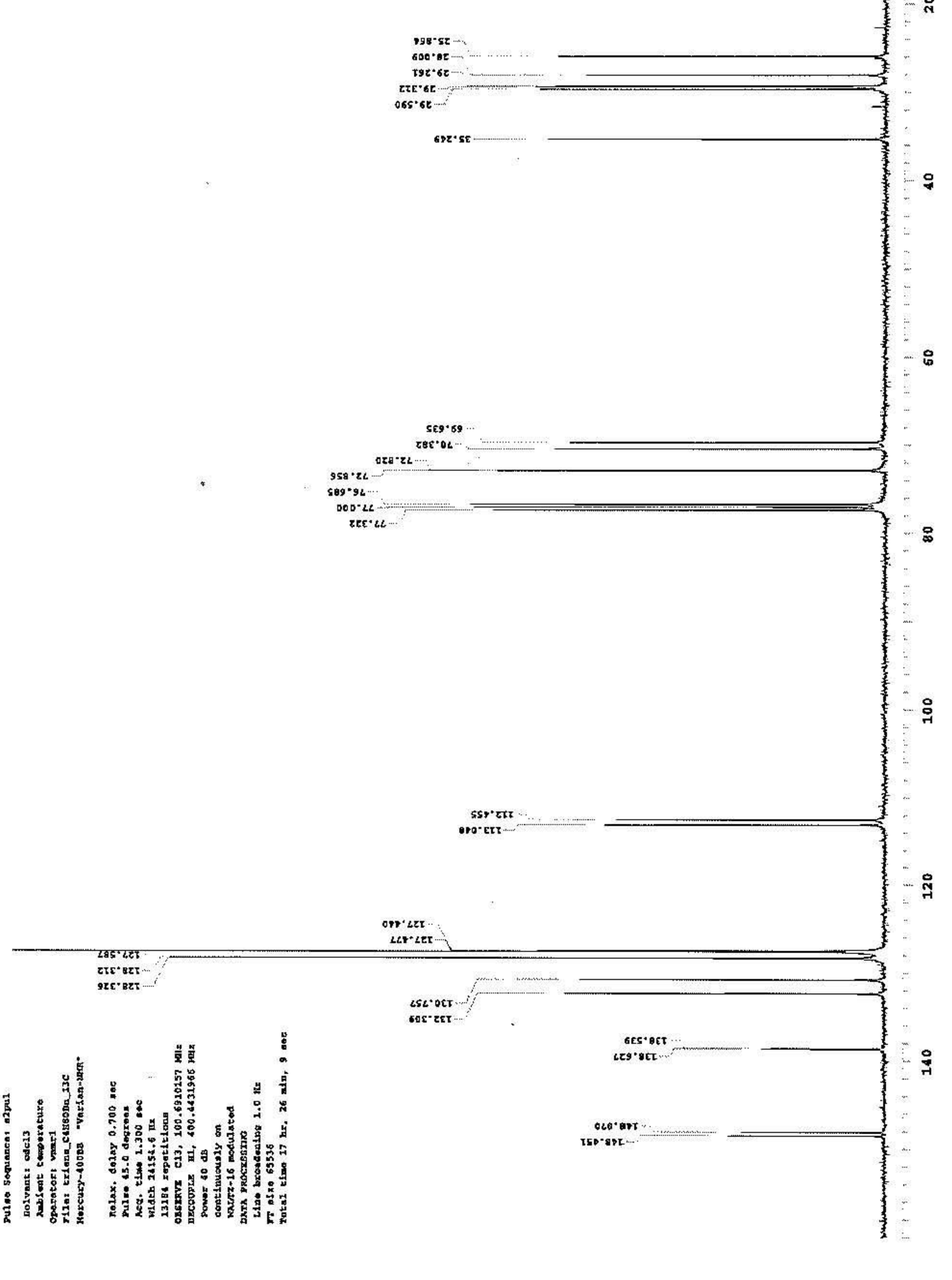

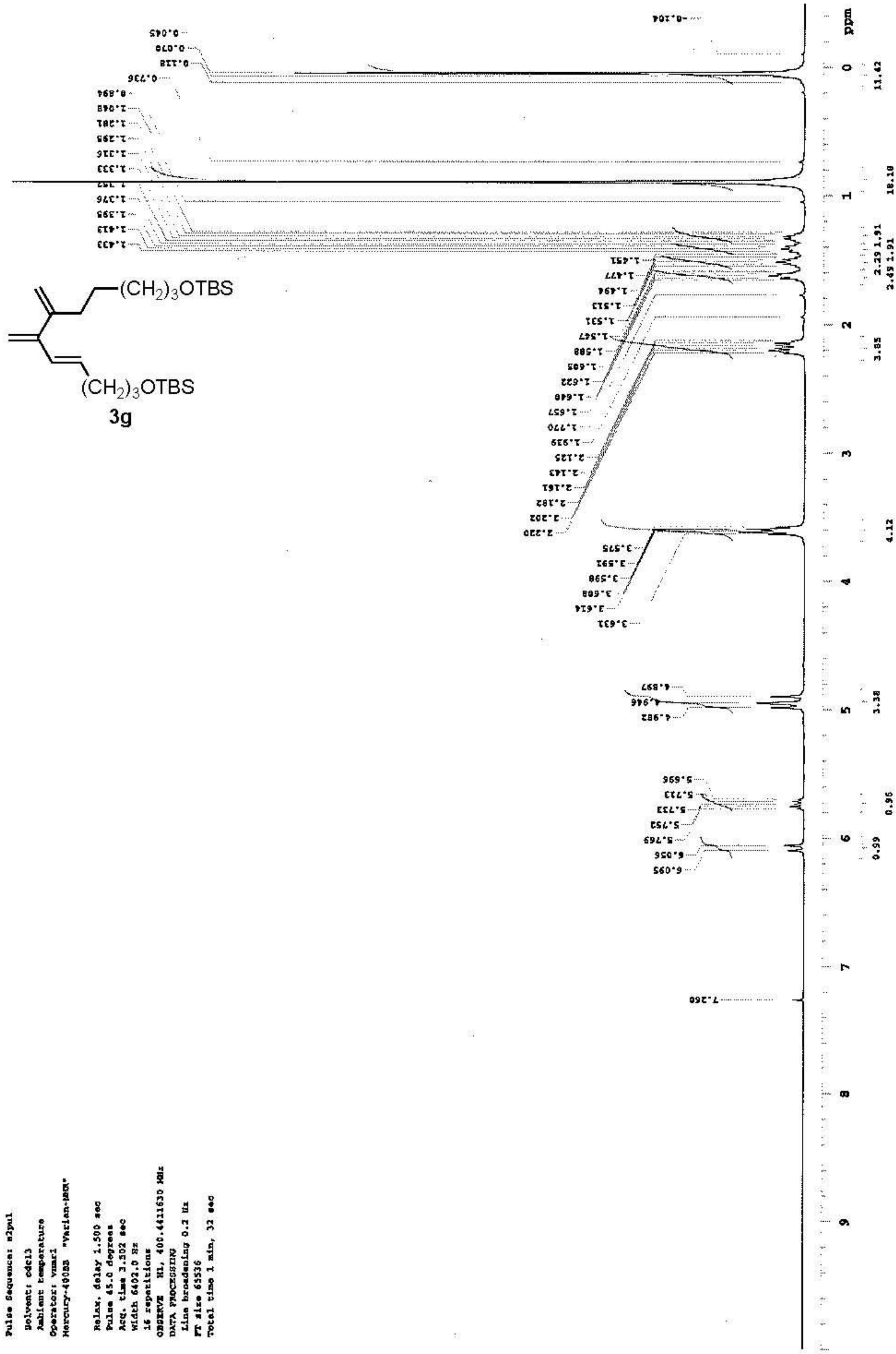

3g

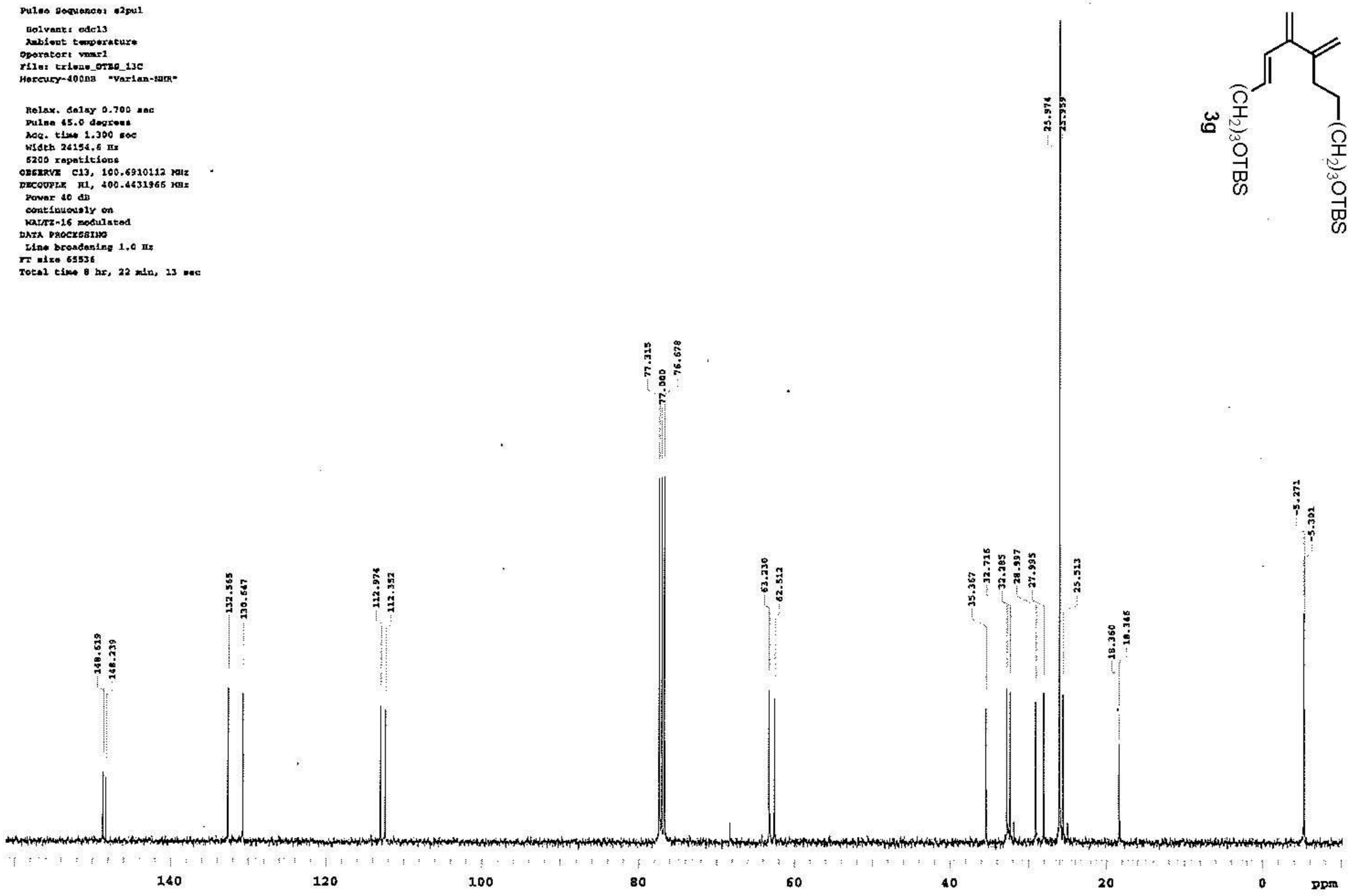
Pulse Sequence: s2pul
Solvent: cdcl3
Ambient temperature
Operator: vnmr1
File: triene_OTBS_13C
Mercury-400BB "Varian-NMR"
Relax. delay 0.700 sec
Pulse 45.0 degrees
Acq. time 1.300 sec
Width 24154.6 Hz
5200 repetitions
OBSERVE C13, 100.6910112 MHz
DECOUPLE H1, 400.4431965 MHz
Power 40 dB
continuously on
WALTZ-16 modulated
DATA PROCESSING
Line broadening 1.0 Hz
FT size 65536
Total time 8 hr, 22 min, 13 sec
148.519
148.239
132.565
130.647
112.974
112.352
77.315
77.000
76.678
63.230
62.512
35.367
32.716
32.285
28.997
27.995
25.974
25.959
25.513
18.360
18.346
-5.271
-5.301
140
120
100
80
60
40
20
0
ppm
(CH2)3OTBS
(CH2)3OTBS

3g

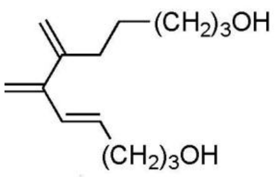
(CH2)3OH
(CH2)3OH

3h

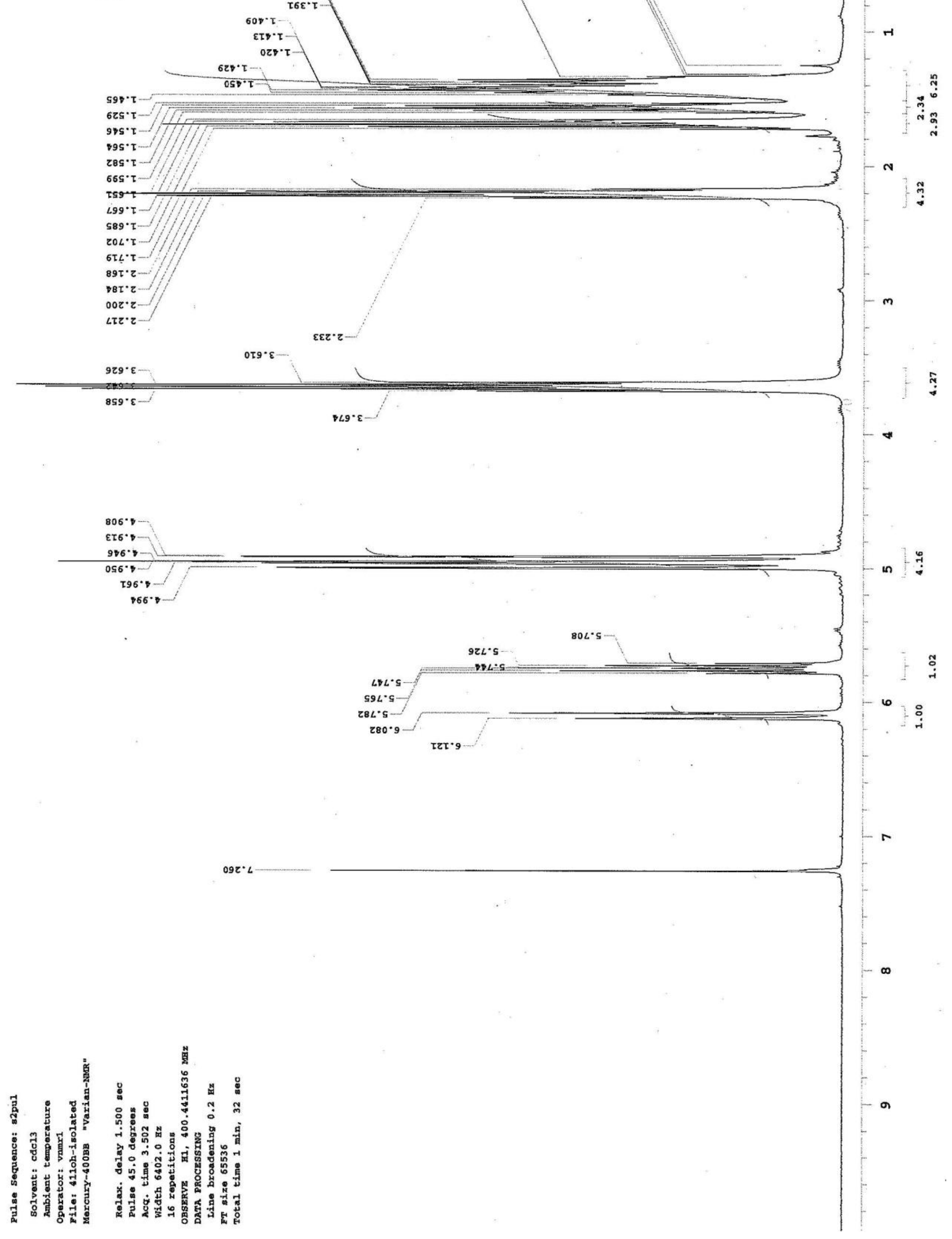
Pulse Sequence: s2pul
Solvent: cdcl3
Ambient temperature
Operator: vnmr1
File: 411oh-isolated
Mercury-400BB "Varian-NMR"
Relax. delay 1.500 sec
Pulse 45.0 degrees
Acq. time 3.502 sec
Width 6402.0 Hz
16 repetitions
OBSERVE H1, 400.4411636 MHz
DATA PROCESSING
Line broadening 0.2 Hz
FT size 65536
Total time 1 min, 32 sec
ppm

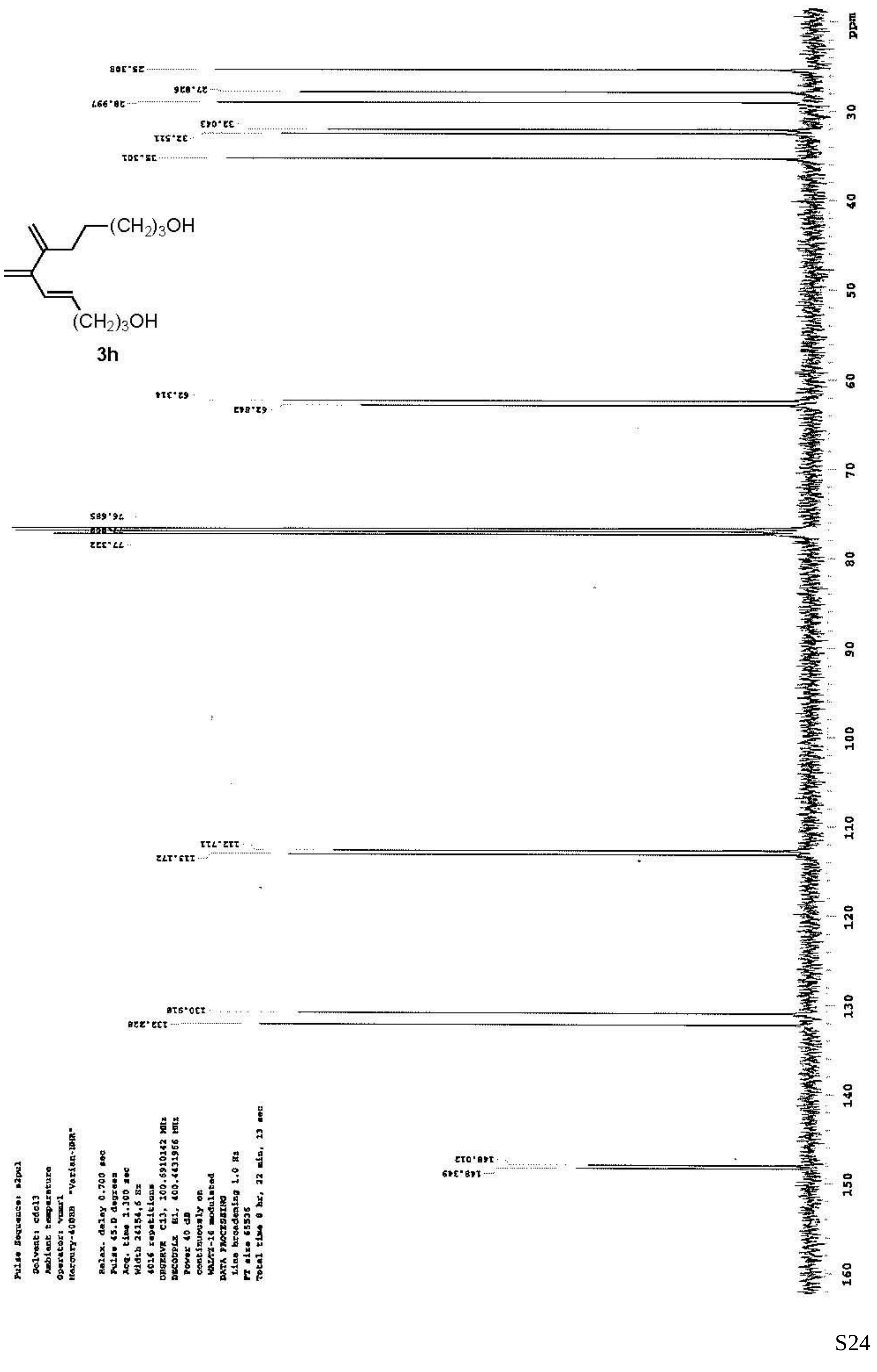
(CH2)3OH
(CH2)3OH
3h
Pulse Sequence: s2pul
Solvent: cdcl3
Ambient temperature
Operator: vnmr1
Mercury-400BB "Varian-NMR"
Relax. delay 0.700 sec
Pulse 45.0 degrees
Acq. time 1.300 sec
Width 24154.6 Hz
4016 repetitions
OBSERVE C13, 100.6910142 MHz
DECOUPLE H1, 400.4431966 MHz
Power 40 dB
continuously on
WALTZ-16 modulated
DATA PROCESSING
Line broadening 1.0 Hz
FT size 65536
Total time 0 hr, 32 min, 13 sec
25.308
27.836
28.997
32.043
32.511
35.301
62.314
62.843
76.685
77.008
77.332
112.711
113.172
130.918
132.328
148.013
148.349
ppm
30
40
50
60
70
80
90
100
110
120
130
140
150
160

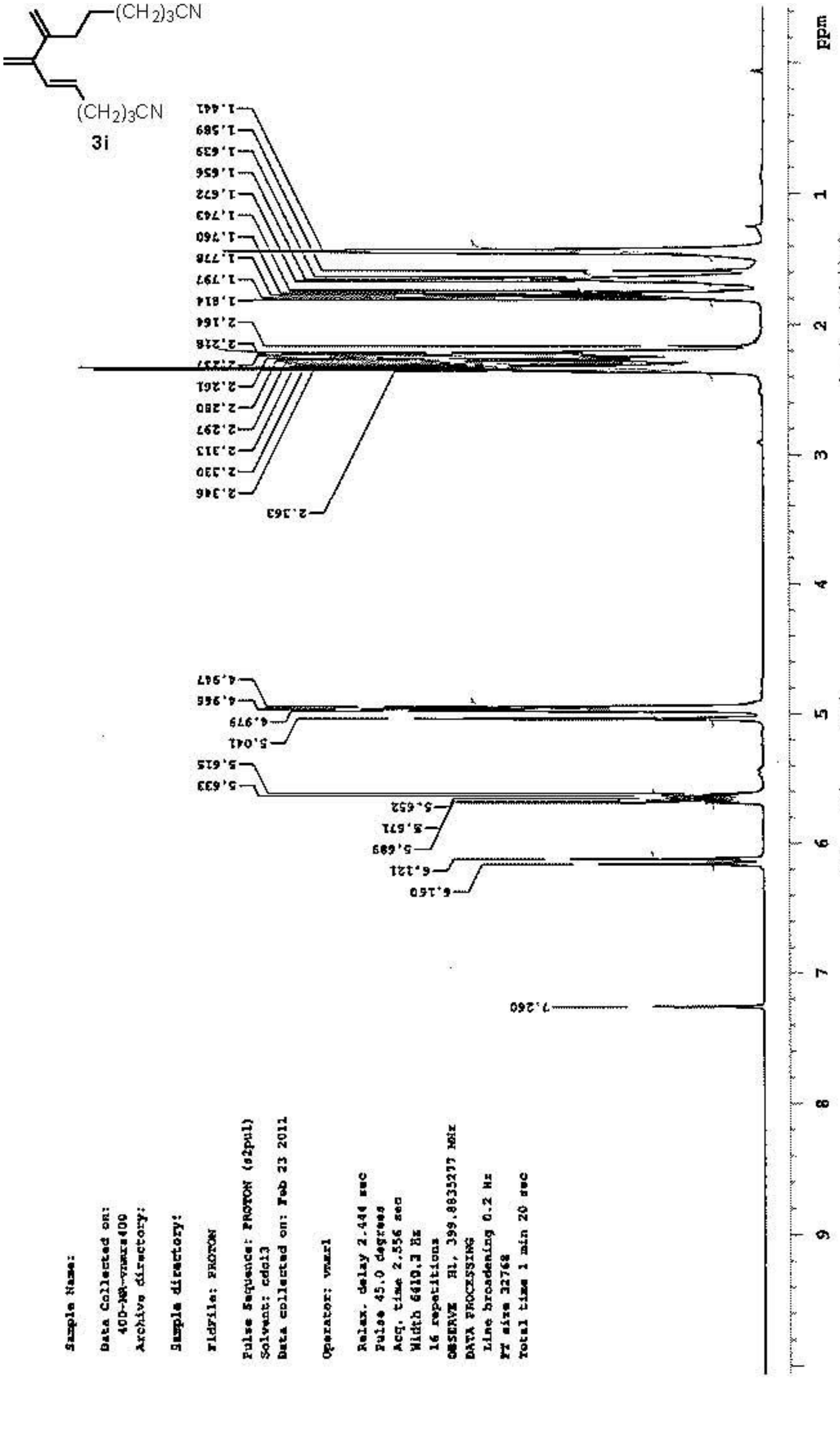
(CH2)3CN
(CH2)3CN
3i
Sample Name:
Data Collected on:
400-MR-vnmrs400
Archive directory:
Sample directory:
FidFile: PROTON
Pulse Sequence: PROTON (s2pul)
Solvent: cdcl3
Data collected on: Feb 23 2011
Operator: vnmr1
Relax. delay 2.444 sec
Pulse 45.0 degrees
Acq. time 2.556 sec
Width 6410.3 Hz
16 repetitions
OBSERVE H1, 399.8835277 MHz
DATA PROCESSING
Line broadening 0.2 Hz
FT size 32768
Total time 1 min 20 sec
ppm

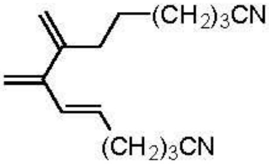
(CH2)3CN
(CH2)3CN

3i

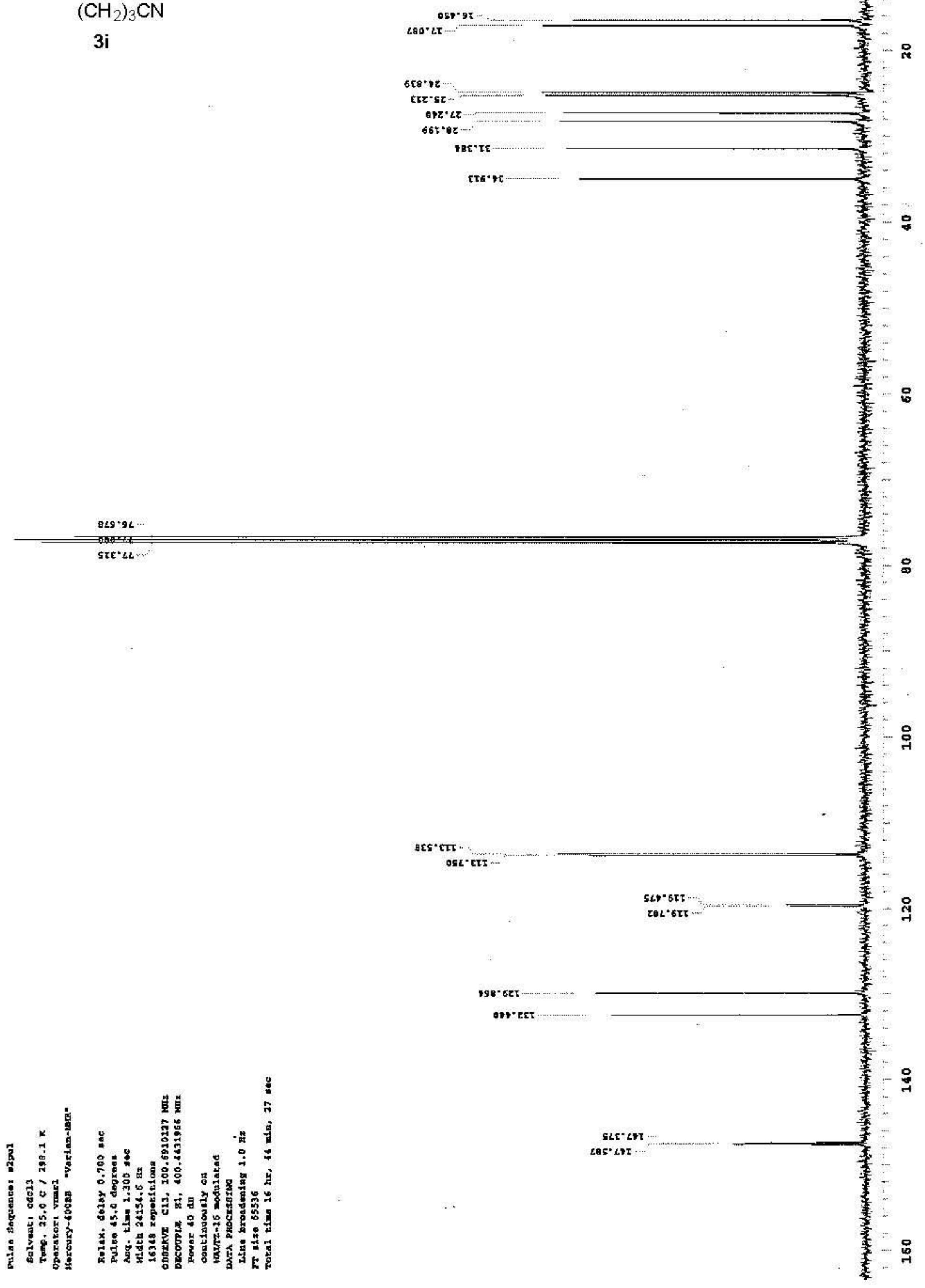
Pulse Sequence: s2pul
Solvent: cdcl3
Temp. 25.0 C / 298.1 K
Operator: vnmr1
Mercury-400BB "Varian-NMR"
Relax. delay 0.700 sec
Pulse 45.0 degrees
Acq. time 1.300 sec
Width 24154.6 Hz
16348 repetitions
OBSERVE C13, 100.6910127 MHz
DECOUPLE H1, 400.4431966 MHz
Power 40 dB
continuously on
WALTZ-16 modulated
DATA PROCESSING
Line broadening 1.0 Hz
FT size 65536
Total time 16 hr, 44 min, 27 sec
16.450
17.087
24.839
25.233
27.248
28.199
31.384
34.913
76.678
77.000
77.315
113.538
113.750
119.475
119.782
129.864
133.440
147.375
147.587
ppm
20
40
60
80
100
120
140
160

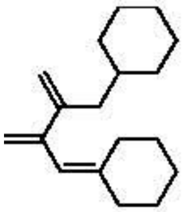

3j

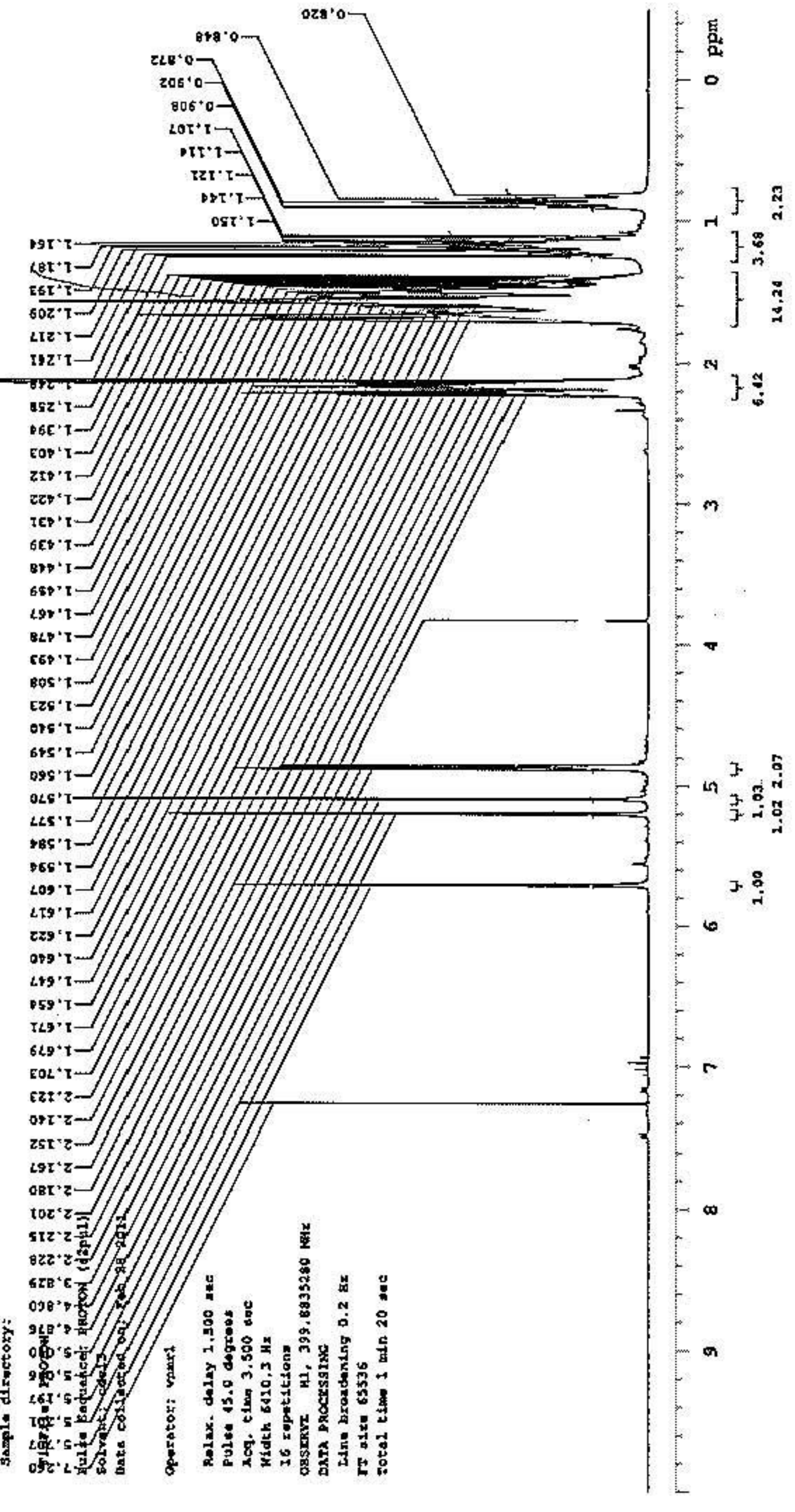

Sample Name:
Data Collected on:
400-MR-vnmrs400
Archive directory:
Sample directory:
FidFile: PROTON
Pulse Sequence: PROTON (s2pul)
Solvent: cdcl3
Operator: vnmr1
Relax. delay 1.500 sec
Pulse 45.0 degrees
Acq. time 3.500 sec
Width 6410.3 Hz
16 repetitions
OBSERVE H1, 399.8835280 MHz
DATA PROCESSING
Line broadening 0.2 Hz
FT size 65536
Total time 1 min 20 sec
0.820
0.848
0.872
0.902
0.908
1.107
1.114
1.121
1.144
1.150
1.164
1.187
1.193
1.209
1.217
1.241
1.248
1.258
1.394
1.403
1.412
1.422
1.431
1.439
1.448
1.459
1.467
1.478
1.493
1.508
1.523
1.540
1.549
1.560
1.570
1.577
1.584
1.594
1.607
1.617
1.622
1.640
1.647
1.654
1.671
1.679
1.703
2.123
2.140
2.152
2.167
2.180
2.201
2.215
2.228
4.860
4.876
5.197
7.260
1.00
1.02
1.03
2.07
6.42
14.24
3.68
2.23
9
8
7
6
5
4
3
2
1
0 ppm

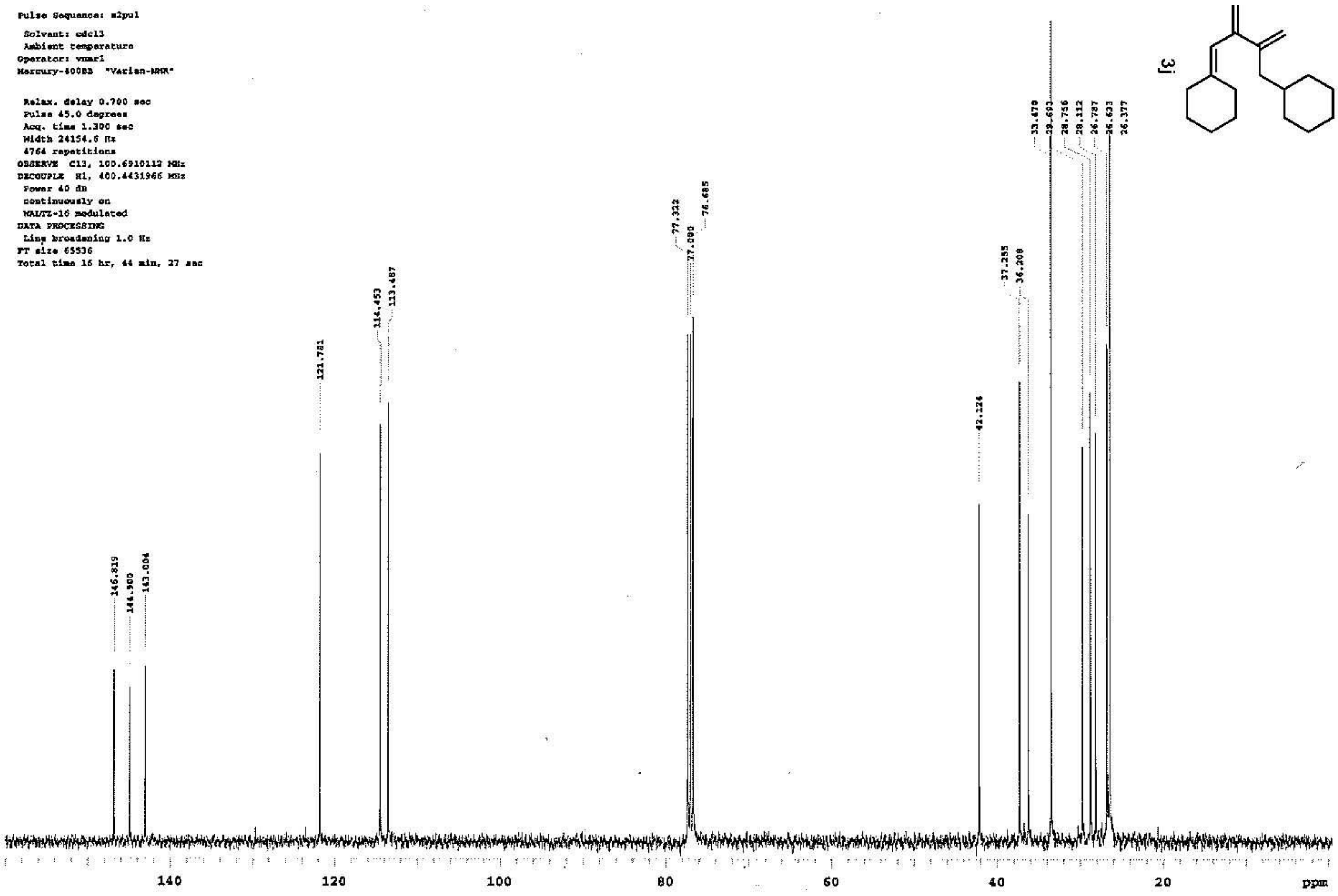

Pulse Sequence: s2pul
Solvent: cdcl3
Ambient temperature
Operator: vnmr1
Mercury-400BB "Varian-NMR"
Relax. delay 0.700 sec
Pulse 45.0 degrees
Acq. time 1.300 sec
Width 24154.6 Hz
4764 repetitions
OBSERVE C13, 100.6910112 MHz
DECOUPLE H1, 400.4431966 MHz
Power 40 dB
continuously on
WALTZ-16 modulated
DATA PROCESSING
Line broadening 1.0 Hz
FT size 65536
Total time 15 hr, 44 min, 27 sec
146.819
144.900
143.004
121.781
114.453
113.487
77.332
77.080
76.685
42.124
37.255
36.208
33.470
28.756
28.112
26.787
26.633
26.377
140
120
100
80
60
40
20
ppm

3j

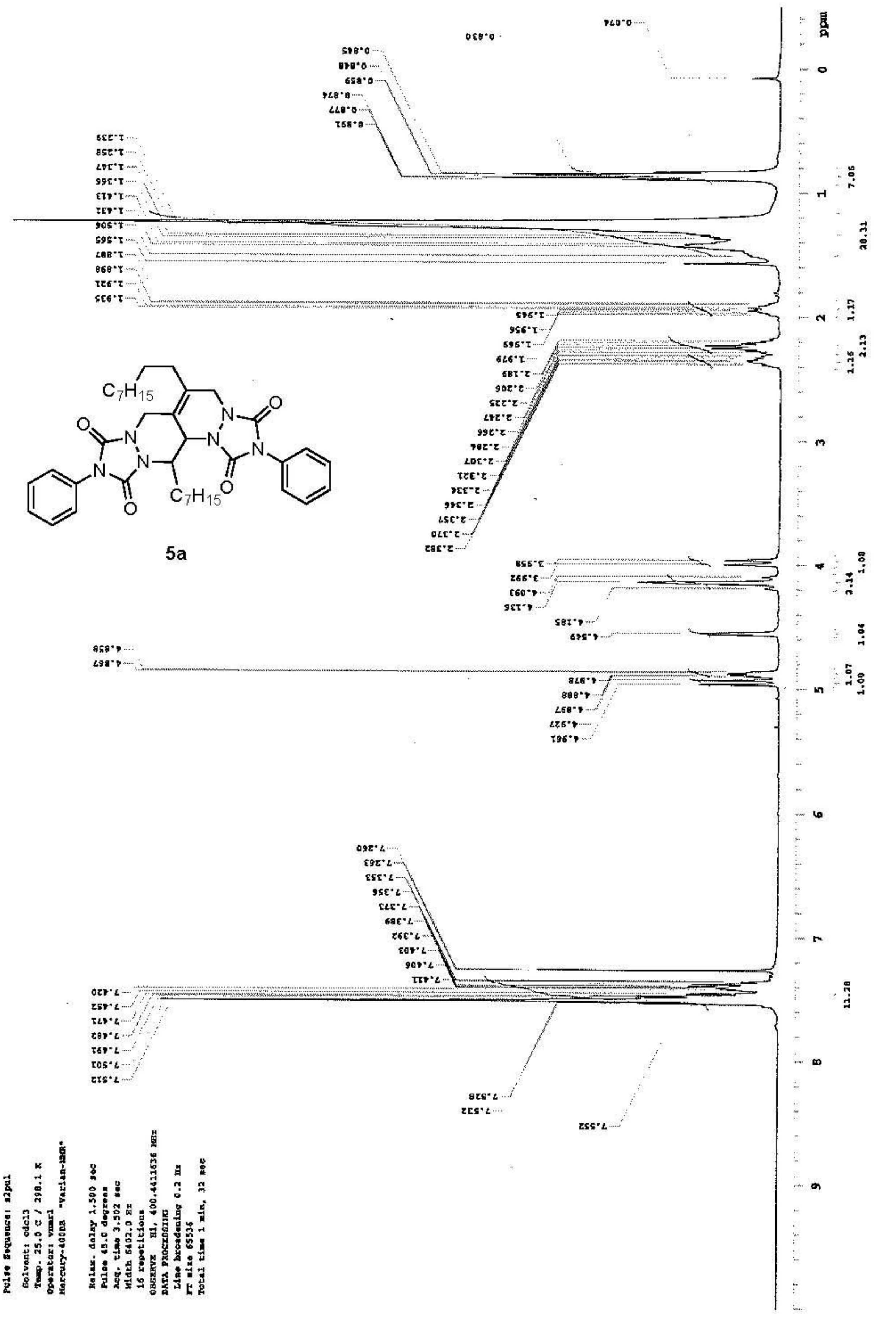

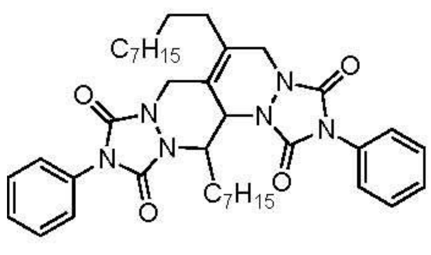

C_7H_{15}
N
O
C_7H_{15}

5a

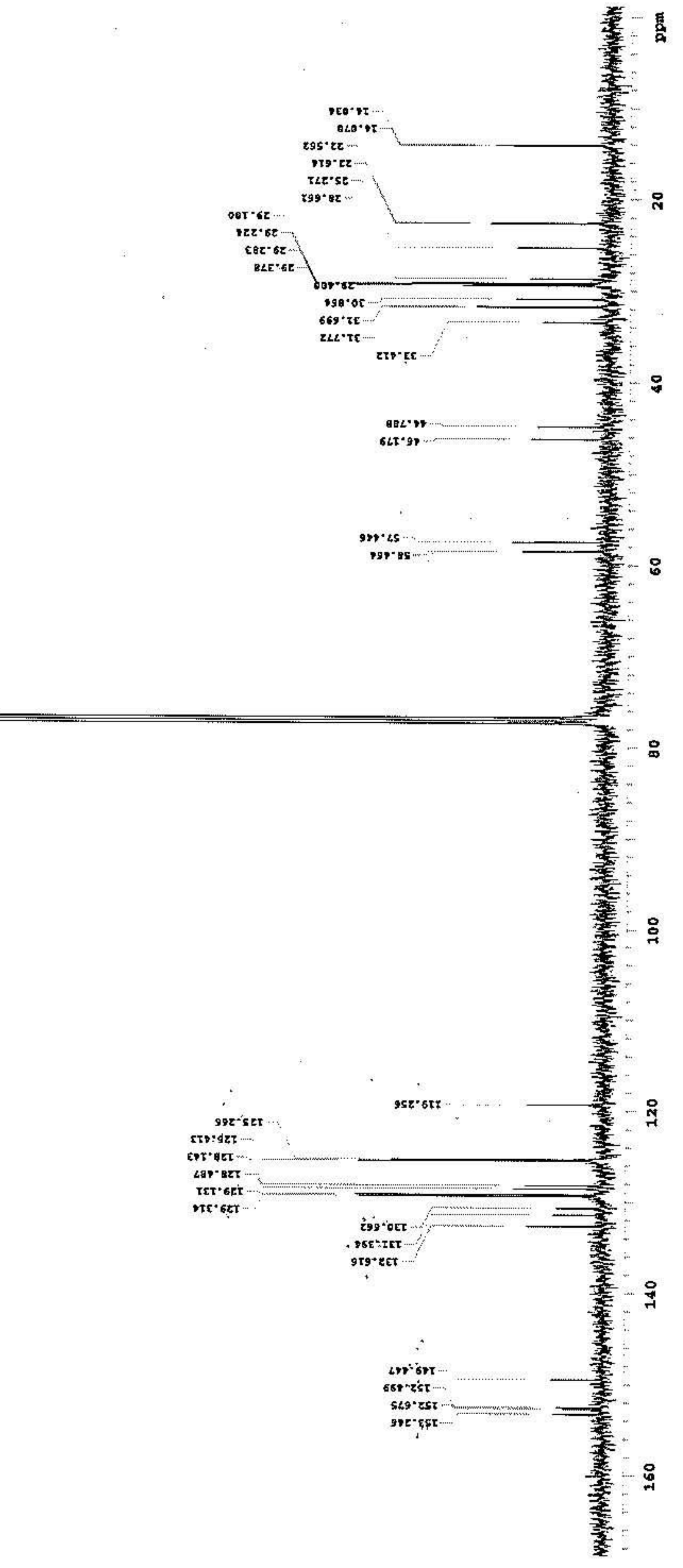

Pulse Sequence: s2pul
Solvent: cdcl3
Temp. 25.0 C / 298.1 K
Operator: vnmr1
File: triazoline_2ke_major_13C
Mercury-400BB "Varian-NMR"
Relax. delay 0.700 sec
Pulse 45.0 degrees
Acq. time 1.300 sec
Width 24154.6 Hz
4056 repetitions
OBSERVE C13, 100.6910120 MHz
DECOUPLE H1, 400.4431966 MHz
Power 40 dB
continuously on
WALTZ-16 modulated
DATA PROCESSING
Line broadening 1.0 Hz
FT size 65536
Total time 16 hr, 44 min, 27 sec
ppm
20
40
60
80
100
120
140
160

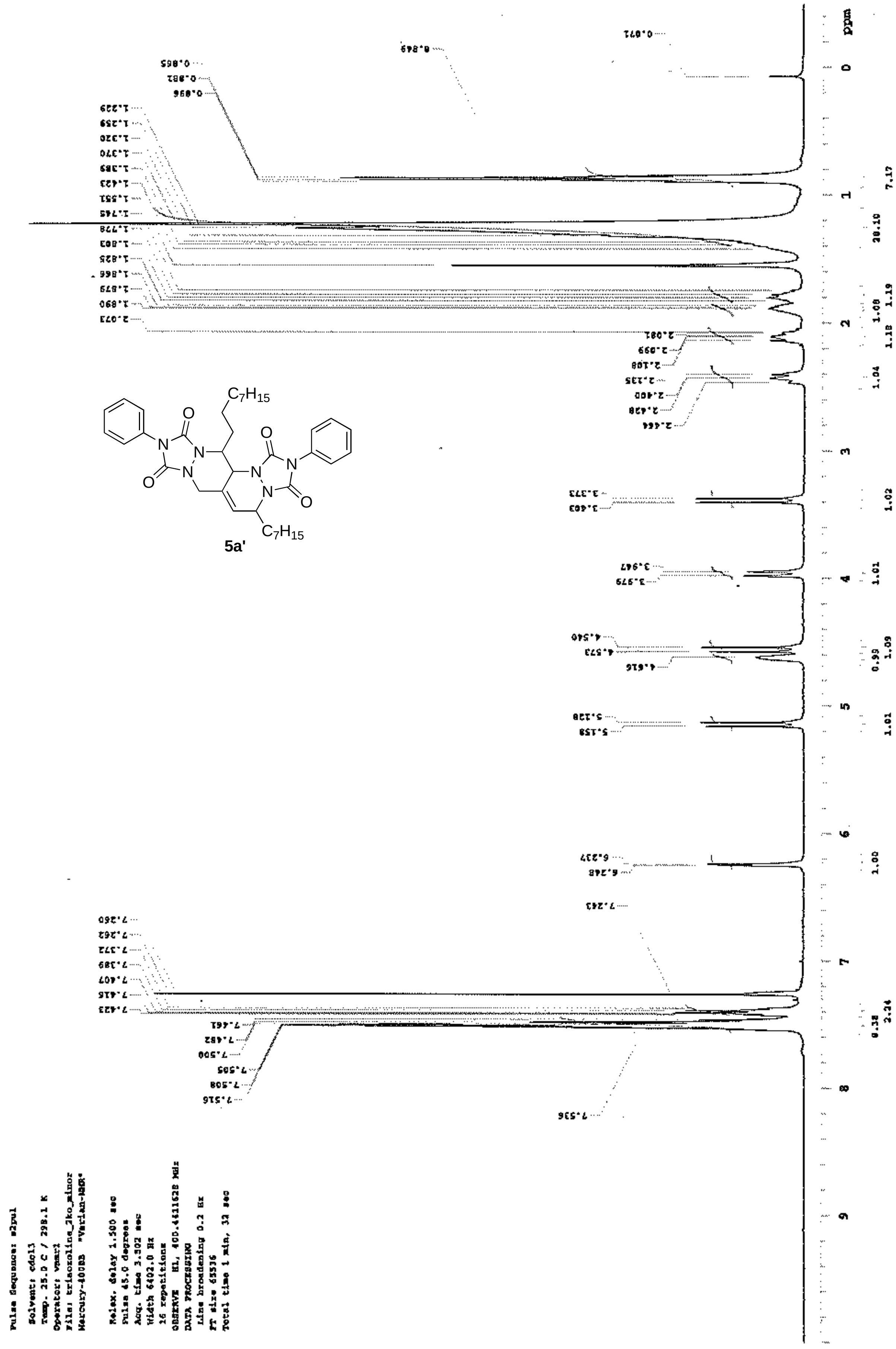
5a'

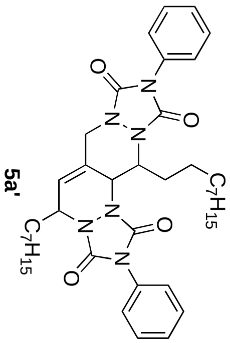

5a'

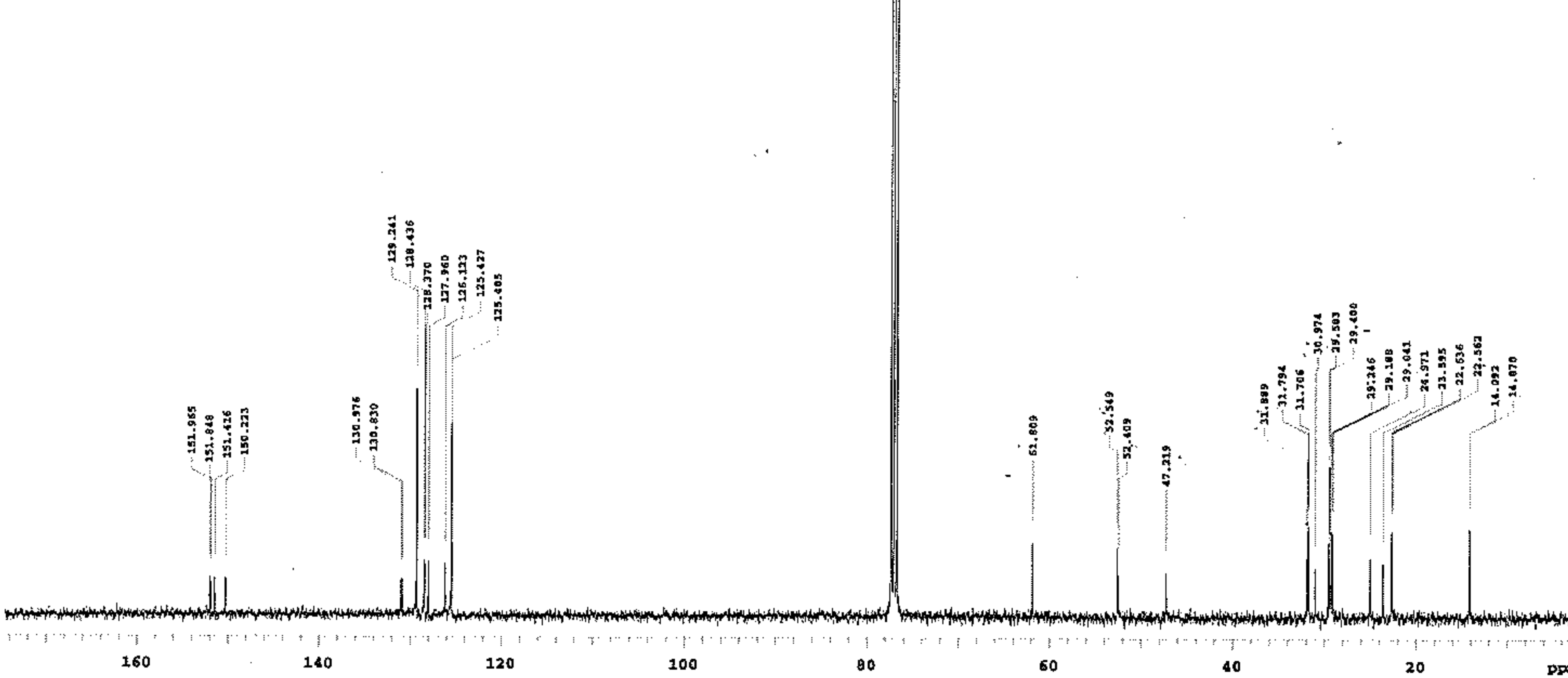

Pulse Sequence: s2pul
Solvent: cdcl3
Ambient temperature
Operator: vmarl
File: triazoline_2ke_minor_13C
Mercury-400BB "Varian-NMR"
Relax. delay 0.700 sec
Pulse 45.0 degrees
Acq. time 1.300 sec
Width 24154.6 Hz
13888 repetitions
OBSERVE C13, 100.6910120 MHz
DECOUPLE H1, 400.4431966 MHz
Power 40 dB
continuously on
WALTZ-16 modulated
DATA PROCESSING
Line broadening 1.0 Hz
FT size 65536
Total time 17 hr, 26 min, 9 sec
151.965
151.848
151.426
150.223
130.976
130.830
129.241
128.436
128.370
127.960
126.123
125.427
125.405
77.322
77.205
77.008
76.685
61.809
52.549
52.409
47.219
31.889
31.794
31.706
30.974
29.503
29.400
29.346
29.188
29.041
24.971
23.595
22.636
22.562
14.092
14.070
160
140
120
100
80
60
40
20
ppm

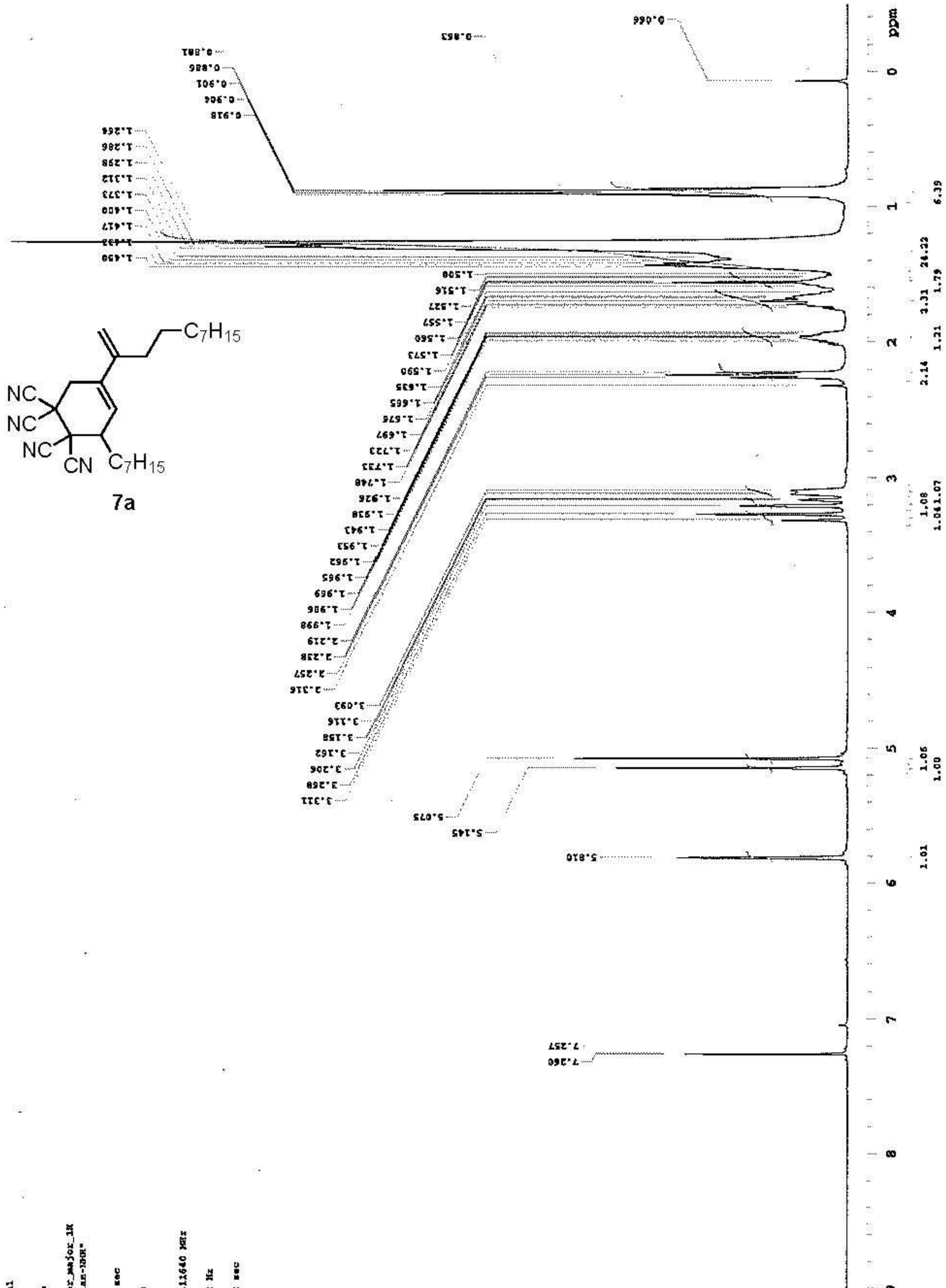

7a

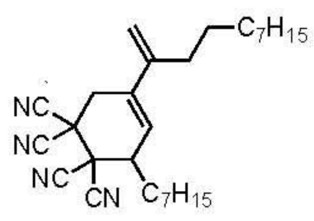

NC
NC
NC
CN
C_7H_{15}
C_7H_{15}

7a

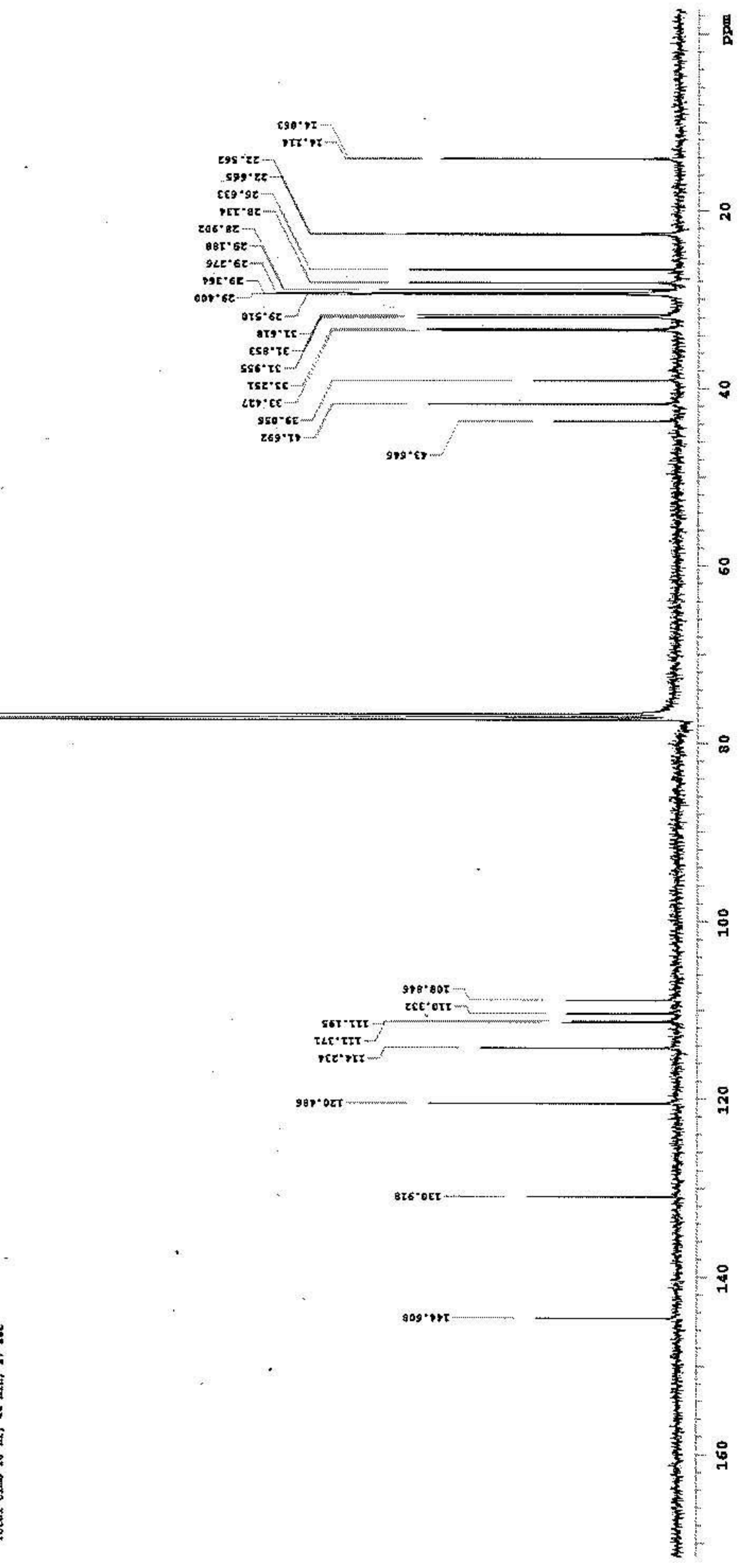

14.063
14.114
22.563
22.665
26.633
28.134
28.902
29.188
29.276
29.364
29.400
29.518
31.618
31.853
31.955
33.351
33.437
39.056
41.692
43.646
76.685
77.000
77.205
77.315
108.846
110.332
111.195
111.371
114.234
120.486
130.918
144.608
ppm
20
40
60
80
100
120
140
160
Pulse Sequence: s2pul
Solvent: cdcl3
Ambient temperature
Operator: vnmr1
Mercury-400BB "Varian-NMR"
Relax. delay 0.700 sec
Pulse 45.0 degrees
Acq. time 1.300 sec
Width 24154.6 Hz
15316 repetitions
OBSERVE C13, 100.6910142 MHz
DECOUPLE H1, 400.4431966 MHz
Power 40 dB
continuously on
WALTZ-16 modulated
DATA PROCESSING
Line broadening 1.0 Hz
FT size 65536
Total time 16 hr, 44 min, 27 sec
